# NLRP3 Regulation in Neonatal Hypoxic–Ischemic Encephalopathy—Focus on Microglial Activation

**DOI:** 10.3390/ijms27177853

**Published:** 2026-09-02

**Authors:** Hannah Burkard, Maria Eugenia Bernis, Anna-Sophie Bremer, Elke Maes, Jonas Walter, Felix Meissner, Hemmen Sabir

**Affiliations:** 1 Department of Neonatology and Pediatric Intensive Care Medicine, University Hospital Bonn, University of Bonn, 53127 Bonn, Germany; 2 Deutsches Zentrum für Neurodegenerative Erkrankungen (DZNE), 53127 Bonn, Germany; 3 Institute of Innate Immunity, Biomedical Center, University Hospital Bonn, BMZ 2, 53127 Bonn, Germany

**Keywords:** newborn brain injury, inflammation, glia cells, neurodevelopmental outcomes, new therapeutic option

## Abstract

Neonatal hypoxic–ischemic encephalopathy (HIE) is a major cause of neonatal mortality and long-term neurological disability, affecting 1–3 per 1000 live births in developed countries and occurring at substantially higher rates in developing countries. Neuroinflammation is a key contributor to disease progression, with growing evidence implicating the activation of the NLR family pyrin domain containing 3 (NLRP3) inflammasome following hypoxic–ischemic (HI) injury. In this study, we investigated the role and regulation of the NLRP3 inflammasome in neonatal HIE using in vitro and in vivo models. Primary microglial cultures subjected to oxygen–glucose deprivation and the Vannucci neonatal rat model of HI were used to characterize NLRP3 activation and its contribution to injury. We demonstrated that HI induces NLRP3 inflammasome activation, whereas pharmacological inhibition of NLRP3 enhances cell viability and attenuates brain damage. Our findings identify microglia as a central mediator of NLRP3-driven neuroinflammation and highlight microglial NLRP3 signaling as a promising therapeutic target for neuroinflammatory diseases. Collectively, this study provides an integrated view of NLRP3 regulation in neonatal HIE and supports inflammasome-directed strategies for neuroprotection following neonatal HI injury.

## 1. Introduction

Neonatal hypoxic–ischemic encephalopathy (HIE) results from impaired cerebral oxygenation and blood flow [1]. The clinical course of HIE ranges from mild to severe, owing to multiple factors that influence disease progression. Affected neonates can suffer from cerebral palsy, cognitive and motor disabilities, and neonatal death [2,3,4].

Cerebral hypoxia forces a shift from aerobic metabolism to anaerobic glycolysis; failure to restore aerobic metabolism prolongs energy failure and results in cell death [1,5,6]. Therapy is required to prevent excessive cell death and brain damage in these patients. Therapeutic hypothermia (TH) is the only treatment for moderate-to-severe HIE. However, there is no reliable therapy for HIE of all severities. In addition to ongoing efforts to develop therapies, research has increasingly focused on elucidating the biological mechanisms that drive post-HIE inflammatory processes.

The importance of inflammatory processes counteracting hypoxia–ischemia (HI) has been demonstrated in animal models of newborn inflammation-sensitized hypoxic–ischemic brain injury. Inflammation worsens the outcome of the pathology and reduces the efficacy of TH [7,8]. Additionally, inflammatory pathways are induced after HI events, even without inflammation pre-sensitization [4,8]. The NLR family pyrin domain containing 3 (NLRP3) inflammasome plays a pivotal role in orchestrating brain inflammation [9,10].

The NLRP3 pathway can be activated by different stimuli, including damage- and pathogen-associated molecular patterns (DAMPs/PAMPs); and plays a key role in sensing cellular stress. Composed of the sensor NLRP3, the apoptosis-associated speck-like protein containing a CARD (ASC), and the effector caspase-1; the inflammasome mediates the cleavage and activation of IL-1β, IL-18, and the pore-forming protein Gasdermin D (GSDMD), thereby inducing pyroptosis [9,10,11,12]. While regulated, the signaling pathway serves to protect the cells and defeats pathogens. However excessive activation can lead to uncontrolled inflammation and cell death [9,10,11,12,13,14].

Microglia express NLRP3. As mediators of inflammation in the brain, they regulate many inflammatory pathways, including Jak/Stat, NF-κB, and inflammasome signaling [15,16]. During neonatal HI, microglia get activated and adopt an early pro-inflammatory phenotype, releasing pro-inflammatory cytokines [8,17,18]. They communicate with other cells via messenger signals, leading to their activation or cell death [18,19,20].

In HIE, microglia are initially primed towards a pro-inflammatory phenotype, leading to upregulation of the NLRP3 inflammasome and amplification of neuroinflammatory responses that contribute to neuronal injury [8,17,18,20,21,22,23]. Additionally, there have been studies on cell-to-cell interaction demonstrating the communication between microglia and neurons in adult models [24,25,26]. Nonetheless, the mechanisms by which microglial NLRP3 modulates neuronal outcomes in neonatal HIE remain to be elucidated. To comprehend this mechanism and to reveal a possible explanation for neuronal loss and brain damage after HI, we performed experiments to demonstrate the importance of NLRP3 in microglial activation and present a possible target for therapeutic interventions.

## 2. Results

### 2.1. Microglia Viability and Morphology Under OGD When Inhibiting NLRP3

**Figure 1:**
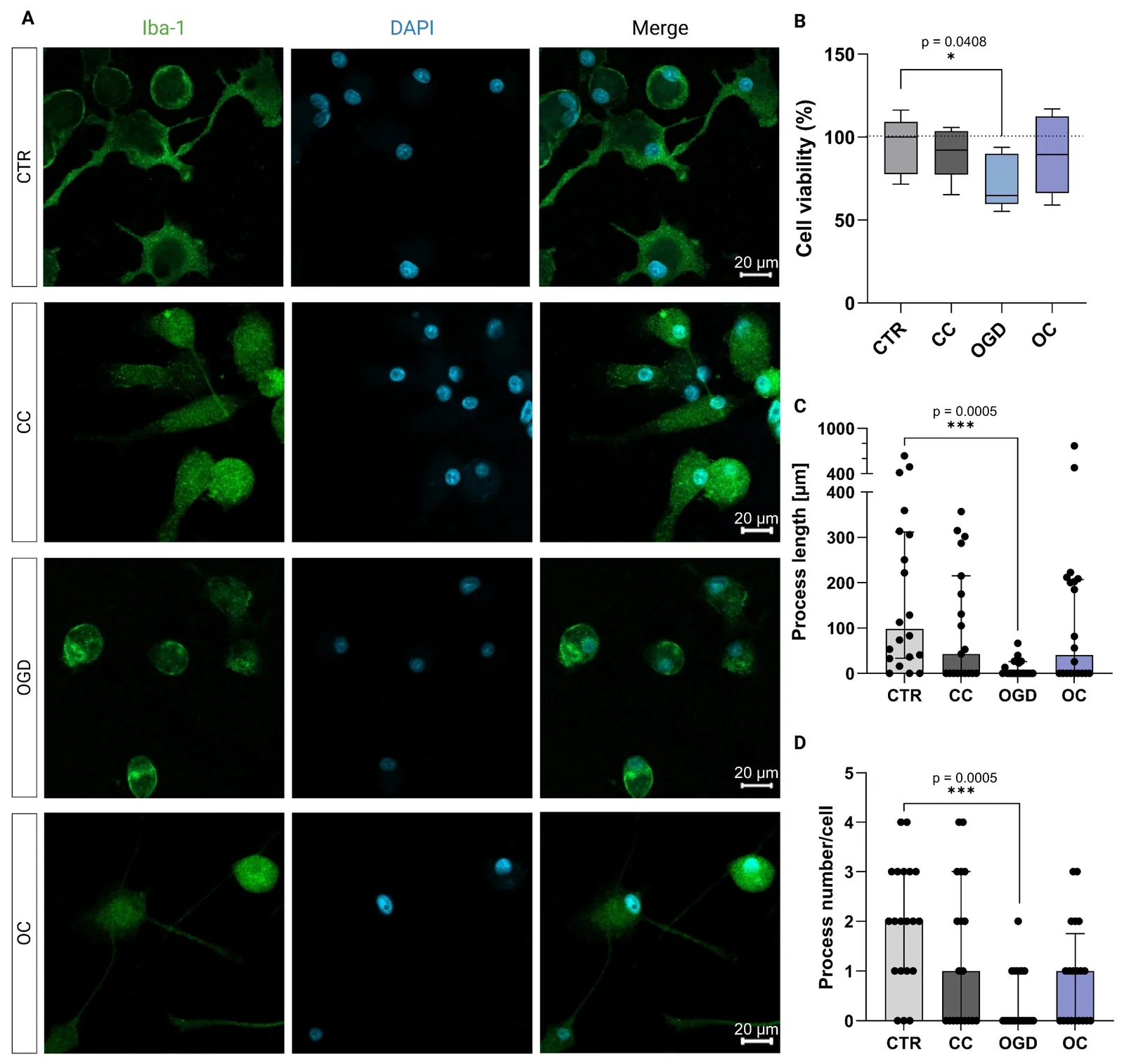
**NLRP3 inhibition during OGD improved microglia cell viability and shifted morphology.** Microglia were exposed to OGD for 6 h at 0.1% O_2_. Thirty minutes before, cells were treated with 1 µM Crid3. CTR = control condition; CC = control + Crid3; OGD = OGD condition; OC = OGD + Crid3. (**A**) Morphology of microglia after different treatments. Cells were incubated under normoxic conditions for 24 h after OGD, fixed with 100% methanol and stained with Iba-1. (**B**) Cell viability assay of microglia treated with OGD and NLRP3 inhibitor Crid3. Three individual MTT assays are presented in boxplots with values normalized to the control median. *N = 4 BR/8 TR.* (**C**,**D**) Analysis of Iba-1-stained microglia. Process number and length were analyzed using Fiji (ImageJ, version 1.54p). Bars represent the median and the interquartile range. *N* = 20 cells (3 BR). Statistics: Kruskal–Wallis-test + Dunn’s multiple comparisons test (* *p* ≤ 0.05 *** *p* ≤ 0.001): (**B**) *H*_4_ = 7.880; (**C**) *H*_4_ = 15.42; (**D**) *H*_4_ = 15.95.

Following OGD, microglial morphology switched from the resting state with a ramified phenotype to the active state with an amoeboid phenotype (Figure 1A). Cells significantly reduced their process length and number (Figure 1C,D). Cell viability decreased significantly after OGD (Figure 1B). The inhibition of NLRP3 with Crid3 was performed in control and OGD-treated cells. Control cells treated with Crid3 showed fewer processes and reduced process length compared to the control; however, cell viability did not change (Figure 1A–D). Cells undergoing OGD and treated with Crid3 showed a rescue of process length and number compared to those undergoing OGD without treatment (Figure 1A,C,D). Cell viability improved when NLRP3 was inhibited in the OGD model (Figure 1A,B). These results indicate a partial rescue of cell viability and morphological changes in microglia under OGD with NLRP3 inhibition.

**Figure 2:**
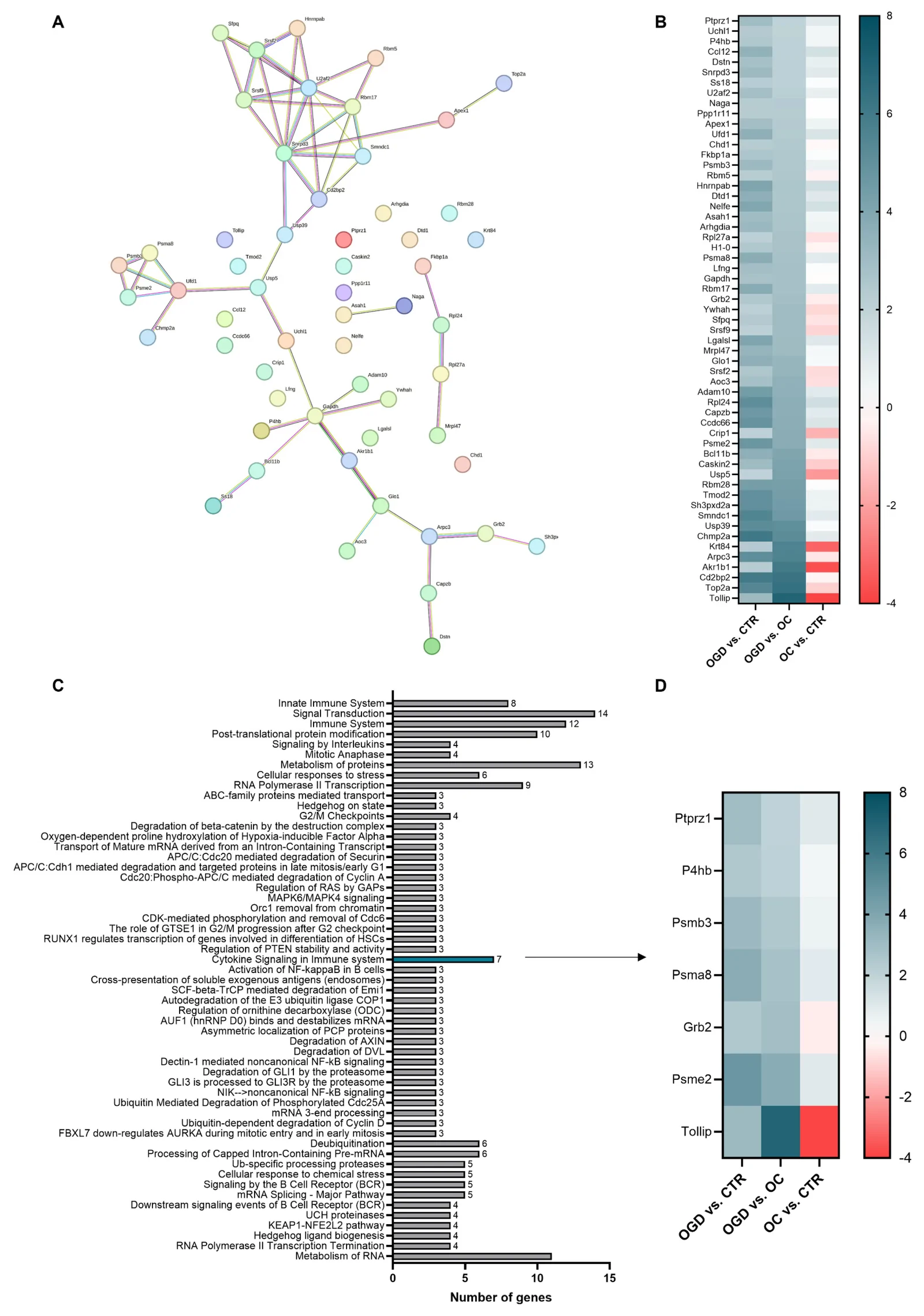
**Microglial protein synthesis specific to OGD-stimulated NLRP3**. Primary microglia were exposed to OGD for 6 h (0.1% O_2_). Cells were treated with 1 µM Crid3 for 30 min before OGD to inhibit NLRP3 (OC). AHA labeling was performed to mark newly synthesized proteins during 24 h of reoxygenation. Supernatant was collected and prepared for MS analysis. (**A**) STRING network of genes specific for NLRP3 activation after OGD (string-db.org). (**B**) Heat map of synthesized genes with displayed log2FC. (**C**) Analysis of reactome pathways using string database (string-db.org). (**D**) Heat map of synthesized genes of reactome pathway cytokine signaling in immune system with displayed log2FC. *N* = 4 *BR.* Differential protein abundance was assessed using limma, fitting linear models with empirical Bayes moderation and correcting *p*-values using the Benjamini–Hochberg FDR (*p*-values ≤ 0.05).

To characterize the microglial secretome associated with NLRP3 activation, conditioned media from primary microglia 24 h after OGD were analyzed using ELISA (Supplementary Figure S2) and unbiased mass spectrometry (Figure 2). Proteomic analysis identified 57 protein-coding genes that were significantly enriched following OGD and normalized by CRID3 treatment, suggesting that their release is influenced by NLRP3-dependent signaling (Figure 2, Supplementary Figure S3 and Supplementary Table S6). Pathway enrichment analysis identified seven proteins involved in cytokine signaling within the immune system: protein tyrosine phosphatase receptor type Z1 (Ptprz1), prolyl 4-hydroxylase subunit beta (P4hb), proteasome subunits (Psmb3, Psma8, and Psme2), growth factor receptor-bound protein 2 (Grb2), and Toll-interacting protein (Tollip) (Figure 2D). Together, these findings indicate that NLRP3 activation influences broader extracellular proteins associated with inflammatory and immune-regulatory pathways.

**Figure 3:**
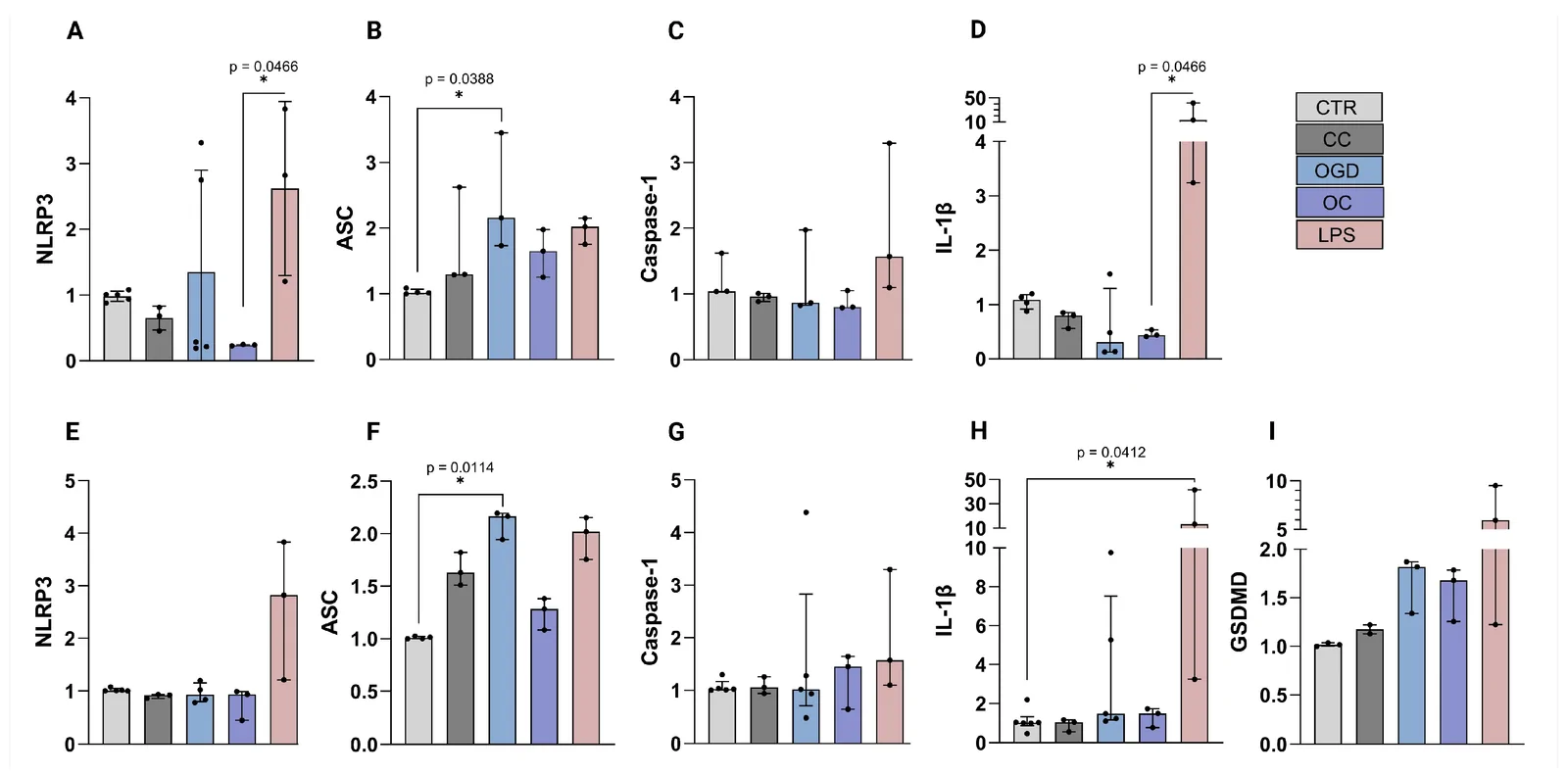
**Transcriptional regulation of the NLRP3 pathway after OGD.** Gene expression of NLRP3 related proteins at 2 h (**A**–**D**) and 12 h (**E**–**I**) following OGD. Gene expression was regulated by NLRP3 inhibition. RT-PCR analysis showed microglia transcription of NLRP3, ASC, Caspase-1, IL-1β and GSDMD after OGD, NLRP3 inhibitor treatment (1 µM Crid3) and LPS treatment (100 ng/mL). CTR = control condition; CC = control + Crid3; OGD = OGD condition; OC = OGD + Crid3; LPS = LPS treatment. Bars represent the median and the interquartile range. *N(CTR/CC/OGD/OC/LPS; BR(TR)): 2 h NLRP3 5(19)/3(9)/5(9)/3(10)/ 3(6)*; *2 h ASC 4(10)/3(7)/3(10)/3(9)/3(6)*; *2 h Caspase-1 3(12)/3(9)/3(9)/3(10)/3(5)*; *2 h IL-1β 4(15)/3(9)/4(13)/3(10)/3(6)*; *12h NLRP3 5(25)/3(10)/4(15)/3(8)/3(6)*; *12 h ASC 4(11)/3(9)/ 3(10)/3(8)/3(6)*; *12 h Caspase-1 5(20)/3(10)/5(15)/3(8)/3(5)*; *12 h IL-1β 6(21)/3(10)/5(16)/3(8)/ 3(6)*; *12 h GSDMD 3(9)/2(6)/3(10)/3(8)/3(5)*. Statistics: Kruskal–Wallis-test + Dunn’s multiple comparisons test (* *p* ≤ 0.05): (**A**) *H*_5_ = 9.373; (**B**) *H*_5_ = 10.49; (**C**) *H*_5_ = 6.633; (**D**) *H*_5_ = 11.35; (**E**) *H*_5_ = 10.61; (**F**) *H*_5_ = 13.72; (**G**) *H*_5_ = 3.419; (**H**) *H*_5_ = 10.72; (**I**) *H*_5_ = 9.924.

After testing different reoxygenation time points following OGD (0, 2, 8, 12, and 24 h), we selected 2 and 12 h for further analysis, as these time points best captured the temporal dynamics of NLRP3 inflammasome activation (Figure 3). At 2 h, the response predominantly involved upstream inflammasome components, particularly NLRP3 and ASC (Figure 3A,B), which was consistent with an early priming response. At 12 h, ASC levels remained elevated, and GSDMD levels showed a later increase (Figure 3F,I). In contrast, changes in downstream components, including caspase-1 and IL-1β, were limited and variable at these time points (Figure 3C,D,G,H). In contrast, the LPS-positive control induced a pronounced downstream transcriptional response, particularly for IL-1β and caspase-1 expression (Figure 3C,D,G,H).

### 2.2. Influence of Activated Microglia on PC12 Cells

To determine whether modulation of microglial NLRP3 signaling influences neuronal injury, PC12 cells were incubated with microglia-conditioned medium (MCM) collected from control, OGD, and OGD+CRID3-treated microglia. Neuronal viability and expression of apoptosis- and inflammation-related markers were assessed to evaluate the impact of the microglial secretome on neuronal survival and inflammatory responses.

**Figure 4:**
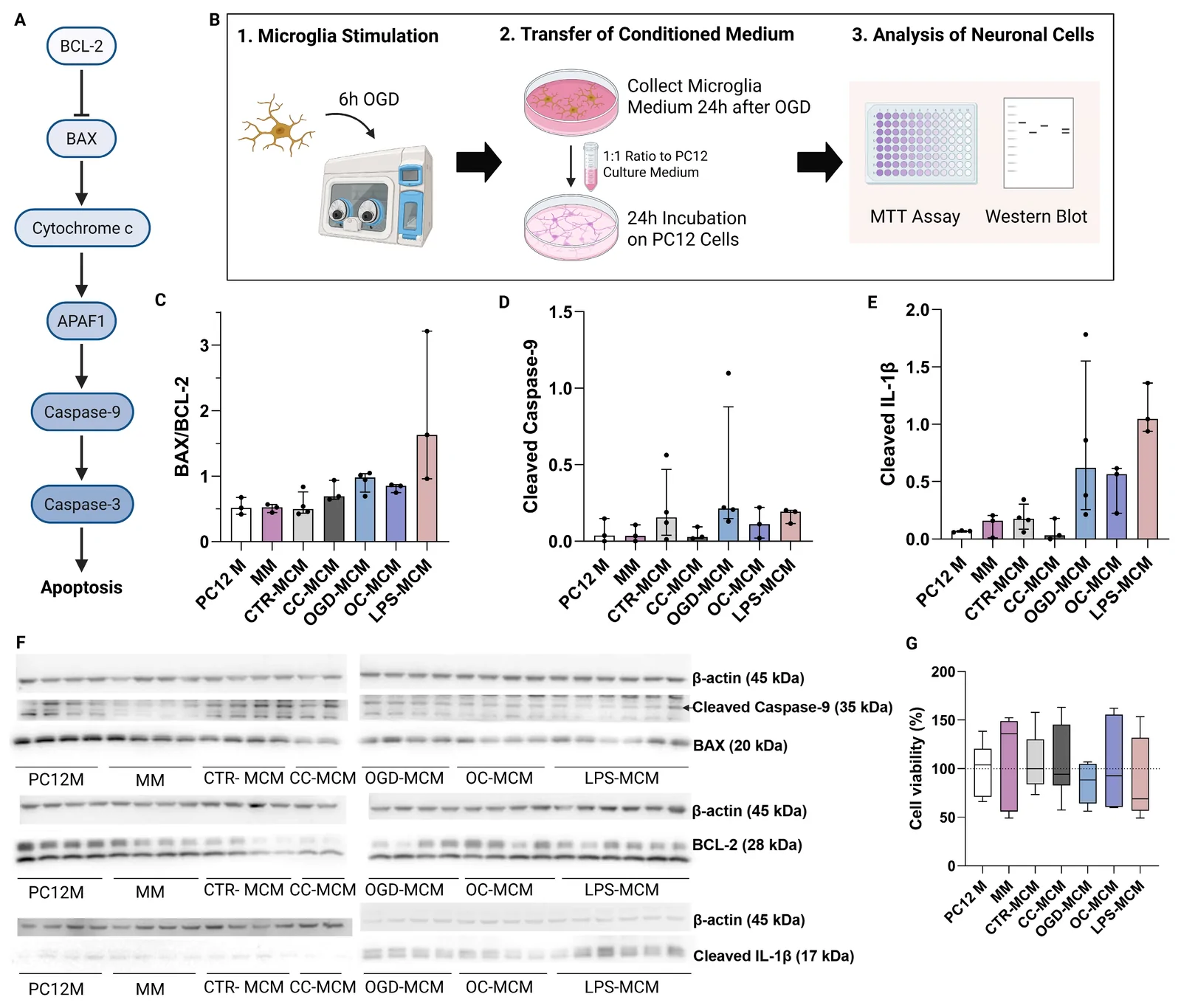
**Effect of microglia-conditioned medium (MCM) on PC12 cells.** (**A**) Apoptotic pathway via BCL-2, BAX and caspase-9. Created using BioRender.com, access date: 1 June 2026. (**B**) Experimental scheme of the conditioned medium experiment. 24 h after microglia stimulation supernatant was collected and incubated on PC12 cells in 1:1 ratio to their culture medium. After 24 h, neuronal cells were analyzed via Western blot and cell viability assay. Created with biorender.com. (**C**–**E**) Protein expression analysis using Western blotting of PC12 cells. Bars display the median and the interquartile range. (**F**) Representative images of the analyzed protein bands used for quantification. Full Western blots in Supplementary Material S11. *N(PC12/MM/CTR/CC/OGD/OC/LPS; BR(TR)) = 3(10)/3(10)/4(16)/3(10)/4(16)/3(10)/3(6).* (**G**) Cell viability assay of PC12 cells treated with MCM. Values are presented in boxplots and normalized to the median of CTR-MCM (*N* = 6). Controls: PC12 M = culture medium of PC12 cells, MM = microglia culture medium. MCM (24 h reoxygenation after OGD experiment (determined with pilot experiments)): control microglia (CTR-MCM), control microglia with Crid3 (CC-MCM), microglia after OGD (OGD-MCM), microglia after OGD with Crid3 (OC-MCM), microglia treated with LPS (LPS-MCM). Statistics: Kruskal–Wallis-test + Dunn’s multiple comparisons test (**C**) *H*_7_ = 16.94; (**D**) *H*_7_ = 10.62; (**E**) *H*_7_ = 17.27; (**G**) *H*_7_ = 2.567.

The protein expression of the apoptosis markers BAX, BCL-2, and caspase-9 showed an increasing trend when PC12 cells were treated with OGD-MCM compared to CTR-MCM. These results indicate that microglial activation leads to the release of apoptosis-inducing factors receivable by neurons (Figure 4C,D). Similarly, cleaved IL-1β in PC12 cells showed the same expression trend when treated with OGD-MCM (Figure 4E).

The viability of neuronal cells did not change significantly in those treated with conditioned medium (Figure 4G).

### 2.3. NLRP3 Inhibition In Vivo Was Dependent on Crid3 Concentration and Injection Time Point

**Figure 5:**
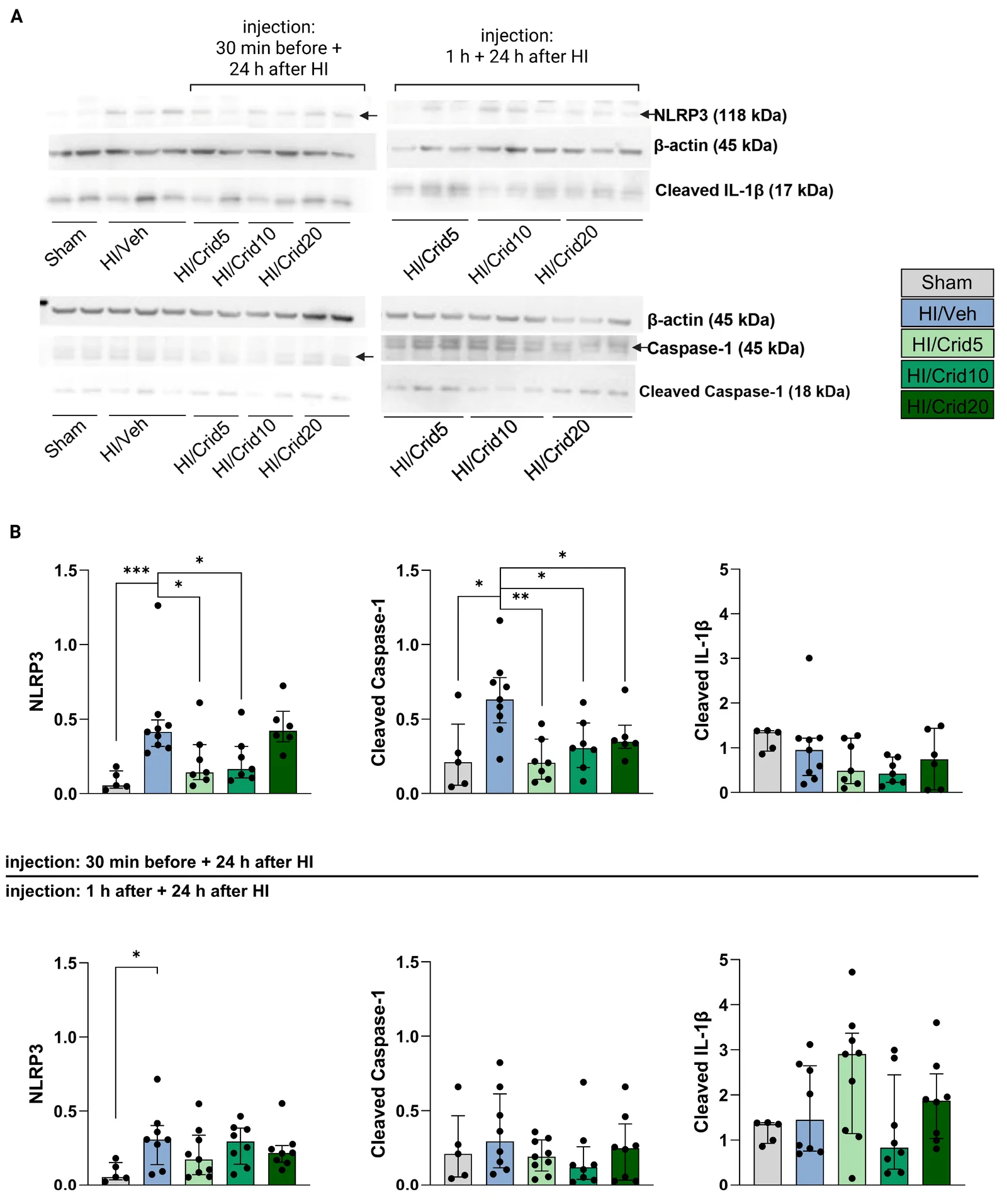
**Comparison of different treatment conditions for inhibiting NLRP3 in an animal model of neonatal HIE**. Protein expression of NLRP3 pathway proteins was analyzed at 48 h after HI in the ipsilateral cortex. (**A**) Representative images of the analyzed protein bands used for quantification. Full Western blots in Supplementary Material S11. (**B**) Protein expression of NLRP3, caspase-1 and IL-1β comparing control animals (Sham, grey bars) vs. hypoxia–ischemia/normothermia-treated animals injected with vehicle (HI/Veh, blue bars) vs. hypoxia–ischemia/normothermia-treated animals injected with 5/10/20 mg/kg Crid3 (HI/Crid5/10/20, green bars). Graphs are separated by different injection times with the upper part showing injections at 30 min before and 24 h after HI and the lower part at 1 h after and 24 h after HI. Bars display the median and the interquartile range. *N* (*Sham*) = 5; *N* (*HI/Veh*) = 8–9; *N* (*HI/Crid5*) = 7–9; *N* (*HI/Crid10*) = 7–8; *N* (*HI/Crid20*) = 6–8. Statistics: Kruskal–Wallis-test + Dunn’s multiple comparisons test (* *p* ≤ 0.05; ** *p* ≤ 0.01; *** *p* ≤ 0.001): (**B**) (**top**) *H*_5_ = 17.02/14.18/7.141; (**B**) (**bottom**) *H*_5_ = 9.259/3.204/5.708.

Injection of the NLRP3 inhibitor Crid3 at different time points (30 min before and 24 h after HI or 1 h and 24 h after HI) and with different doses (5/10/20 mg/kg) showed diverse effects on the efficiency of NLRP3 inhibition 48 h after HI. The treatment parameters were selected based on preliminary experiments (Supplementary Table S1), designed using conditions reported in the literature. The inhibition efficiency was tested by analyzing the protein levels of NLRP3 pathway proteins in the ipsilateral cortex of animals which underwent hypoxic–ischemic injury. Saline was injected at the same time points as Crid3 to serve as a positive control for the effect of HIE in neonatal rats (Figure 5).

NLRP3 and caspase-1 were upregulated in the HI/Vehicle group compared to the Sham group (Figure 5A,B, light blue). Due to the variability of the model, the statistical significance differed between experiments. IL-1β expression did not increase at 48 h after HI (Figure 5A,B). Comparing the different time points of injection and dose used, the data showed that an injection of 5 mg/kg Crid3, 30 min before and 24 h after HI reached the most effective dose–response for NLRP3 inhibition compared to that in the HI/Vehicle group (Figure 5A,B, light green). Particularly, the 5 mg/kg Crid3 dose demonstrated a significant change in the protein expression of cleaved caspase-1, an effector of the inflammasome (Figure 5A,B). Based on these results, 5 mg/kg dose of Crid3 was selected for further analysis.

### 2.4. The Effect of NLRP3 Inhibition onNLRP3 Pathway Proteins After HI

**Figure 6:**
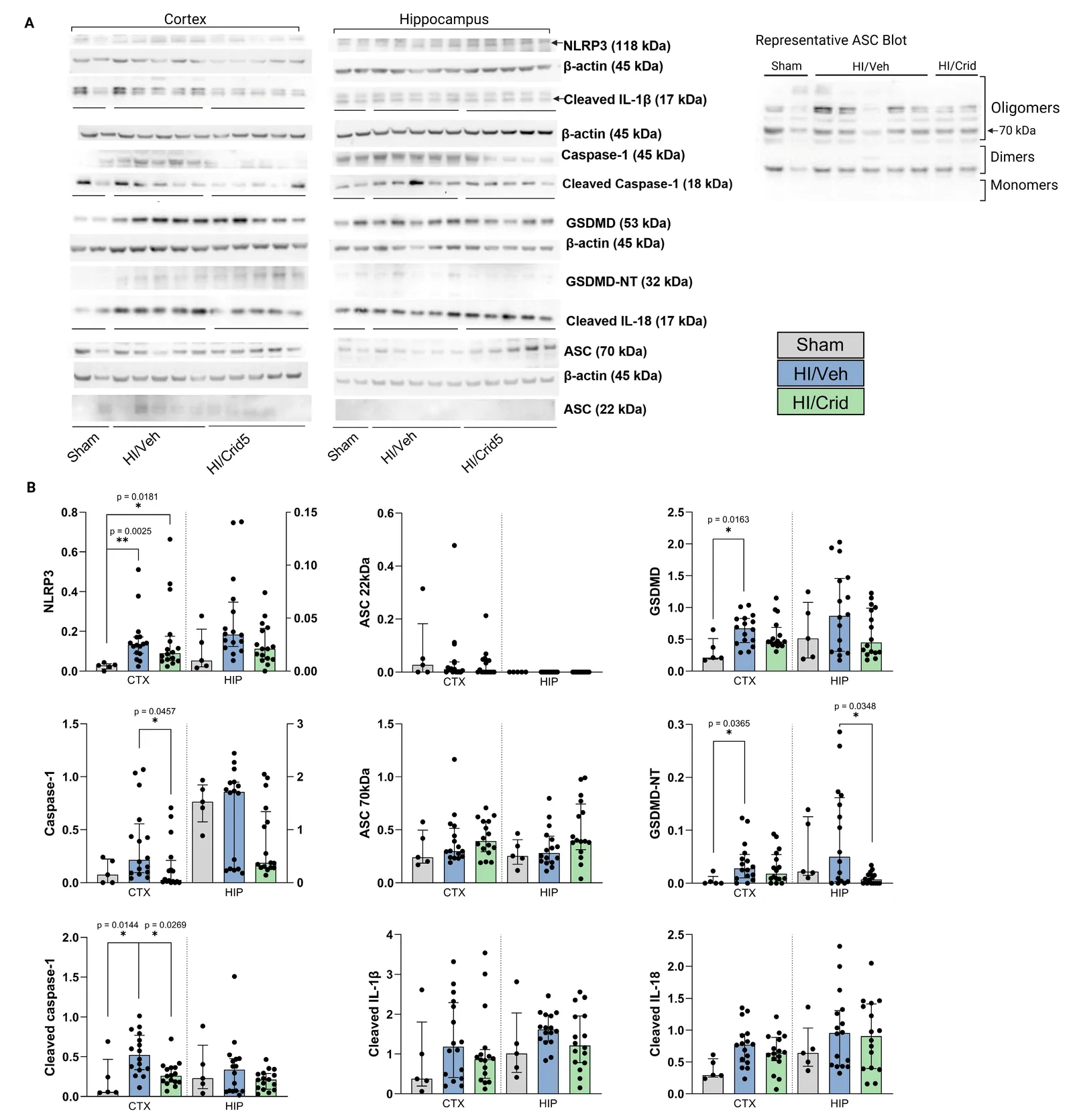
**NLRP3 inhibition affects NLRP3 pathway protein expression 24 h after HI**. Rats were i.p. injected 30 min before and 24 h after HI with 5 mg/kg Crid3 (HI/Crid) or vehicle (HI/Veh). Brain areas were collected 24 h after HI. (**A**) Representative images of the analyzed protein bands used for quantification. Full Western blots in Supplementary Material S11. (**B**) Protein expression of NLRP3 pathway proteins comparing control animals (Sham, grey bars) vs. hypoxia–ischemia/normothermia treated animals injected with vehicle (HI/Veh, blue bars) vs. hypoxia–ischemia/normothermia treated animals injected with 5 mg/kg Crid3 (HI/Crid, light green bars). Ipsilateral cortex (CTX) and hippocampus (HIP) are separated with a dotted line. Bars display the median and the interquartile range. *N* (*Sham*) = 5; *N* (*HI/Veh*) = 16; *N* (*HI/Crid*) = 16. Statistics: Kruskal–Wallis-test + Dunn’s multiple comparisons test (* *p* ≤ 0.05; ** *p* ≤ 0.01): (**B**) NLRP3: *H*_3_ = 11.27/6.128; ASC22 kDa: *H_3_* = 30.88/n.a.; GSDMD: *H*_3_ = 7.733/2.261; Caspase-1: *H*_3_ = 7.175/2.819; ASC70 kDa: *H*_3_ = 1.983/4.558; GSDMD-NT: *H*_3_ = 6.298/8.063; Cleaved caspase-1: *H*_3_ = 11.00/1.060; IL-18: *H*_3_ = 5.433/0.6779; IL-1β: *H*_3_ = 1.620/3.844.

NLRP3 pathway protein expression was analyzed 24 h and 48 h after HI in the ipsilateral cortex and hippocampus. At 24 h after treatment, HI/Vehicle showed a significant upregulation of NLRP3, cleaved caspase-1, GSDMD, and GSDMD-NT in the cortex, whereas the same increase but not significant was observed in the hippocampus. The protein ASC as monomer (22 kDa) and oligomer (70 kDa) remained unchanged (Figure 6B).

Treatment with 5 mg/kg Crid3 efficiently inhibited the upregulation of caspase-1, cleaved caspase-1, and GSDMD-NT (Figure 6A,B).

**Figure 7:**
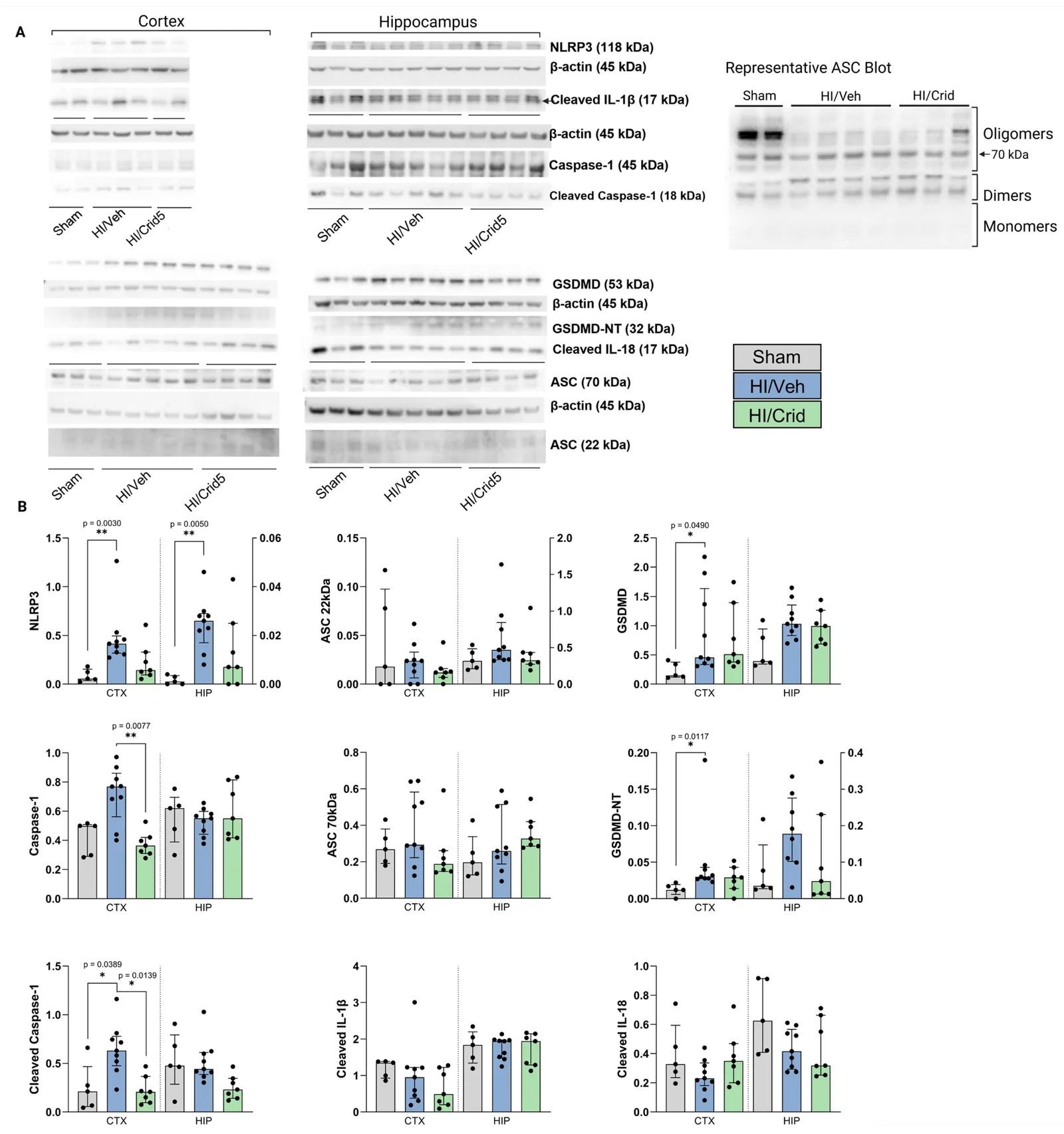
**NLRP3 inhibition affects NLRP3 pathway protein expression 48 h after HI**. Rats were i.p. injected at 30 min before and 24 h after HI with 5 mg/kg Crid3 (HI/Crid) or vehicle (HI/Veh). Brain areas were collected at 48 h after HI. (**A**) Representative images of the analyzed protein bands used for quantification. Full Western blots in Supplementary Material S11. (**B**) Protein expression of NLRP3 pathway proteins comparing control animals (Sham, grey bars) vs. hypoxia–ischemia/normothermia-treated animals injected with vehicle (HI/Veh, blue bars) vs. hypoxia–ischemia/normothermia-treated animals injected with 5 mg/kg Crid3 (HI/Crid, light green bars). Ipsilateral cortex (CTX) and hippocampus (HIP) are separated with a dotted line. Bars display the median and the interquartile range. *N* (*Sham*) = 5; *N* (*HI/Veh*) = 9; *N* (*HI/Crid*) = 7. Statistics: Kruskal–Wallis-test + Dunn’s multiple comparisons test (* *p* ≤ 0.05; ** *p* ≤ 0.01): (**B**) NLRP3: *H*_3_ = 11.36/10.25; ASC22 kDa: *H*_3_ = 1.199/3.749; GSDMD: *H*_3_ = 6.568/4.788; Caspase-1: *H*_3_ = 10.43/0.4561; ASC70 kDa: *H*_3_ = 3.365/4.165; GSDMD-NT: *H*_3_ = 8.423/4.051; Cleaved caspase-1: *H*_3_ = 10.23/6.820; IL-18: *H*_3_ = 2.474/3.607; IL-1β: *H*_3_ = 3.763/0.02822.

At 48 h, HI/Vehicle treatment significantly increased NLRP3, cleaved caspase-1, GSDMD, and GSDMD-NT levels in the cortex, while only NLRP3 levels were significantly upregulated in the hippocampus (Figure 7A,B). Crid3 reduced the upregulation of these proteins and significantly lowered the expression of cleaved caspase-1 (Figure 7A,B). The interleukins did not show any increase after HI. As interleukins are regulated by many different pathways and their expression is time-dependent, expression patterns may differ at other time points.

The results validated the NLRP3-dependent upregulation of pathway proteins and the effect of the chosen treatment with 5 mg/kg Crid3. The cortex and hippocampus showed similar trends, with the cortex expressing higher levels of NLRP3 protein and showing significant protein upregulation due to HI (Figure 6 and Figure 7).

### 2.5. Transcription of NLRP-3-Related Genes in Microglia After HI

**Figure 8:**
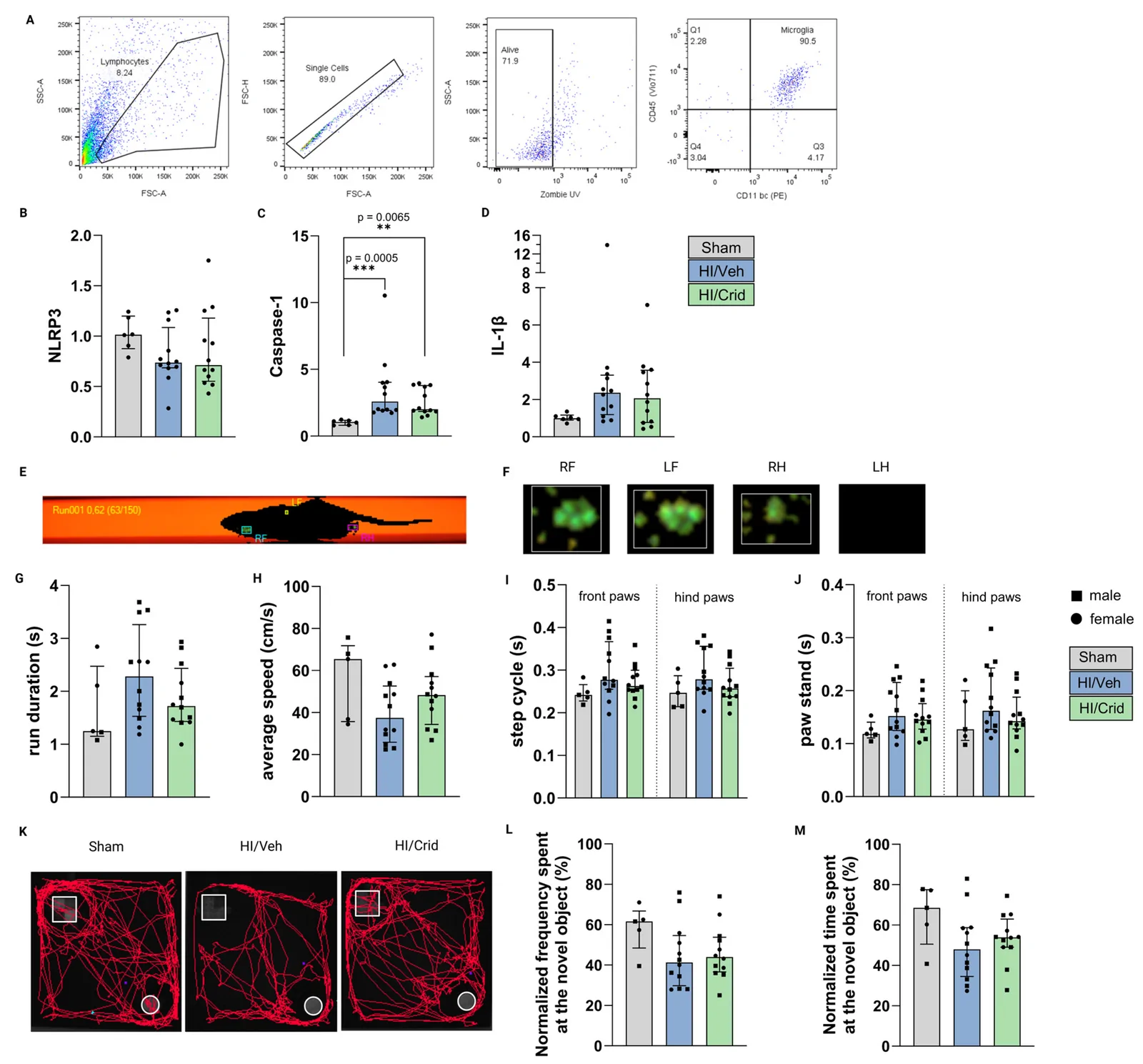
**Effect of NLRP3 inhibition on microglial transcription and long-term outcome in the animal.** Treatment groups: Sham, HI/Vehicle and HI/Crid (5 mg/kg Crid3). (**A**–**D**) Transcription of NLRP3 pathway proteins in isolated cortical microglia 24 h after neonatal HIE. (**A**) Flow cytometry gates used to determine microglia CD11b/c^+^-CD45^+^ cells. (**B**–**D**) NLRP3 pathway-related genes analyzed using RT-PCR. *N* (*Sham*) = 6, *N* (*HI/Veh*; *HI/Crid*) = 12. (**E**–**M**) Animals were tested using Catwalk test for motor function assessment at P48 (**E**–**J**) and novel object recognition (NOR) for cognitive function at P76 (**K**–**M**). (**E**) Exemplary image of a rat walking through the CatWalk setup. (**F**) Exemplary images of the right front (RF), left front (LF), right hind (RH) and left hind (LH) paws. (**G**–**J**) Analysis of run duration, average speed, step cycle and paw stand. Bars represent the median and the interquartile range of the averages of three runs per animal. (**K**) Exemplary image of recorded movements of rats in the NOR setup. The white square represents the novel object, the white circle represents the old object. (**L**,**M**) Analysis of frequency and time discovering the novel object normalized to the total contact with both objects. Bars represent the median and the interquartile range. Males (squares); females (circles). *N* (*Sham*) = 5, *N* (*HI/Veh*, *HI/Crid*) = 12. Statistics: Kruskal–Wallis-test + Dunn’s multiple comparisons test (** *p* ≤ 0.01; *** *p* ≤ 0.001): (**B**) *H*_3_ = 3.108; (**C**) *H*_3_ = 14.63; (**D**) *H*_3_ = 4.303; (**G**) *H*_3_ = 2.634; (**H**) *H*_3_ = 3.951; (**I**) *H*_3_ = 4.078/3.267; (**J**) *H*_3_ = 3.186/1.598; (**L**) *H*_3_ = 3.113; (**M**) *H*_3_ = 3.643.

Components of the NLRP3 pathway were specifically analyzed in isolated cortical microglia cells via MACS 24 h after HI and quantifying their gene expression using RT-PCR (Figure 8A–D). Microglia purity was determined by flow cytometry. Following exclusion of debris, doublets and dead cells, cells were analyzed for CD45 and CD11b expression. Microglia were defined as CD11b^+^ CD45^+^ and the purity was calculated as the percentage of CD11b^+^ CD45^+^ cells among total live cells. A purity of more than 90% was used for further analysis (Figure 8A). At the gene expression level, NLRP3 was not upregulated 24 h after HI (Figure 8B). The inflammasome effector caspase-1 and its downstream target IL-1β showed increased gene expression in both treatment groups exposed to HI. At this time point, no effect of Crid3 treatment was observed (Figure 8C,D).

### 2.6. NLRP3 Inhibition Affected Motor Behavior Functions After Neonatal HIE

The long-term effects of NLRP3 inhibition were assessed by evaluating the motor and cognitive functions of the animals used in the study. Assessing motor function using Catwalk behavior test, showed that HI led to an increase in run duration, described by a lower speed, longer step cycle, and paw stand, compared to the Sham group. Treatment with Crid3 showed a trend of reducing the run duration induced by HI (Figure 8G–J). For the assessment of cognitive function, novel object recognition (NOR) test was used. Animals from the HI/Vehicle group tended to have reduced interest in the novel object, as quantified by reduced frequency and time spent discovering the novel object. NLRP3 inhibition did not alter the frequency or time of novel object discovery (Figure 8L,M). Our data showed that NLRP3 inhibition could potentially affect motor function in the long term after HI exposure.

### 2.7. Inhibition of NLRP3 Improved Brain Tissue Damage After Neonatal HIE

**Figure 9:**
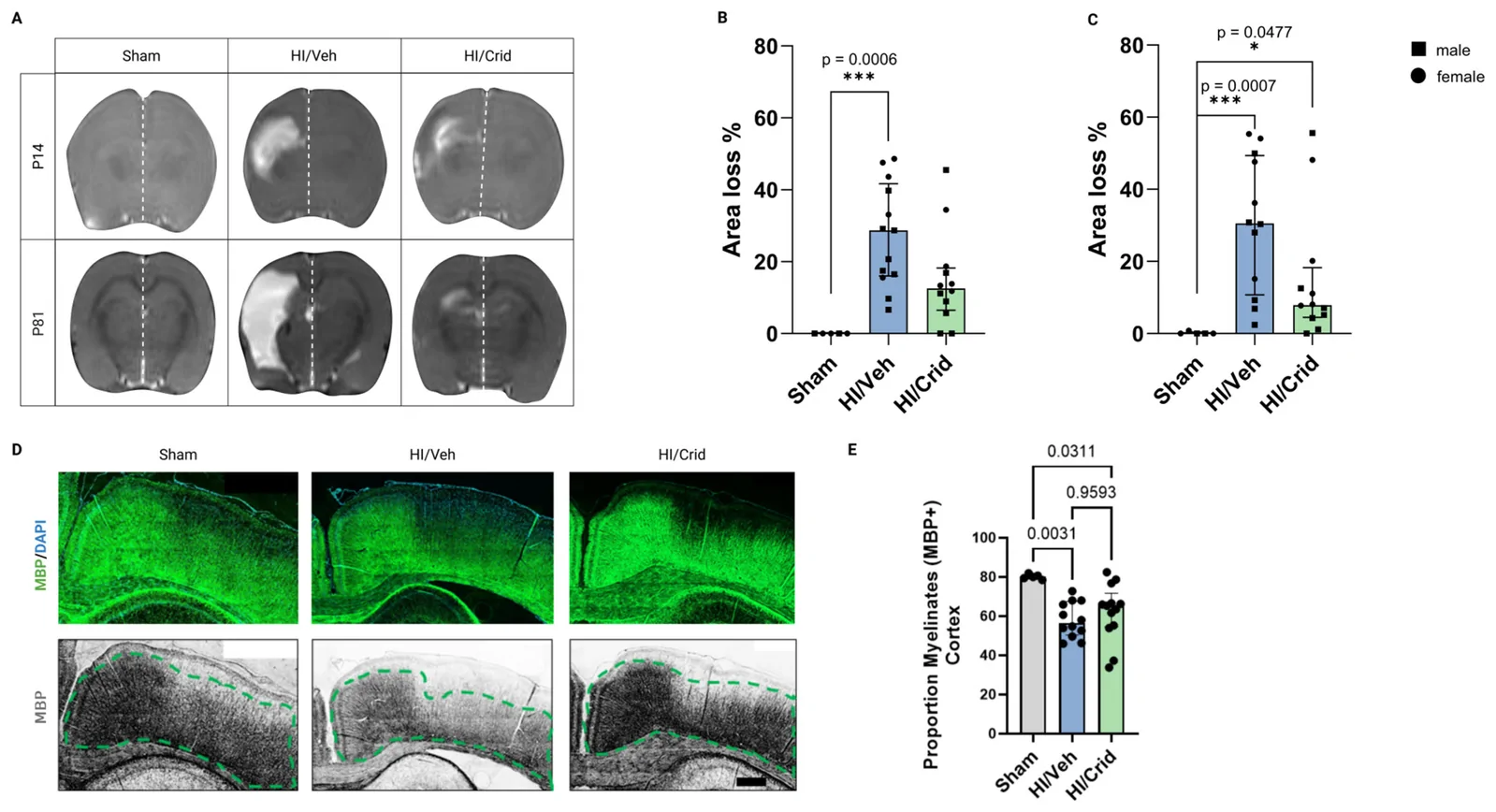
**Effect of NLRP3 inhibition on brain injury.** Treatment groups: Sham, HI/Vehicle and HI/Crid (5 mg/kg Crid3). (**A**) Animals were scanned using MRI at 7/74 days after HI and brain area loss was measured using T2-weighted imaging. Exemplary T2-weighed MRI images of coronal brain sections at Bregma −0.88 mm. Hemispheres are separated with a dotted line. (**B**,**C**) Brain area loss of different treatments at P14 (**B**) and P81 (**C**). (**D**) Exemplary confocal images of the cortical area showing myelin basic protein (MBP) in green and gray with DAPI as a nuclear marker in blue. Dashed outline in the gray images showed the MPB areas quantified. (**E**) Proportion of Myelinated (MBP+) area normalized to the total cortical area. Bars represent the median and the interquartile range. Males (squares); females (circles). *N* (*Sham*) = 5, *N* (*HI/Veh*, *HI/Crid*) = 12. Statistics: Kruskal–Wallis-test + Dunn’s multiple comparisons test (* *p* ≤ 0.05; *** *p* ≤ 0.001): (**B**) *H*_3_ = 14.17; (**C**) *H*_3_ = 13.63.

At P14 and P81, the brains of the animals were scanned using an MRI. Compared with that in the Sham group, the area loss in the HI/Vehicle group was significantly increased (Figure 9B,C). Treatment with the NLRP3 inhibitor Crid3 showed a neuroprotective effect, as the area loss decreased at both time points from a median around 30% (HI/Veh) to approximately 15% (HI/Crid) (Figure 9B,C). No significant differences were observed between the sexes (Supplementary Figure S4). Taken together, the MRI data highlight the importance of NLRP3 in the development of brain damage following HI.

As white matter injury is a major consequence of neonatal HI, cortical myelination was assessed at P81 by immunostaining with myelin basic protein (MBP). Sham animals displayed uniform MBP immunoreactivity throughout the cortex, whereas vehicle-treated HI animals showed a marked reduction in cortical myelination (Figure 9D). In contrast, CRID3-treated animals exhibited substantial preservation of MBP-positive fibers, consistent with improved white matter integrity (Figure 9D). Quantification demonstrated a significant increase in the proportion of myelinated cortex in CRID3-treated animals compared to vehicle-treated HI animals, with values approaching those observed in sham controls (Figure 9E). Together, these findings demonstrate that early pharmacological inhibition of the NLRP3 inflammasome confers durable neuroprotection after neonatal HI by reducing long-term tissue loss and preserving cortical myelination in the brain.

## 3. Discussion

This study aimed to investigate NLRP3 activation in microglia following experimental neonatal HIE and assess its impact on neuronal like cells, elucidating the deleterious role of microglia-mediated NLRP3 activation in HIE-induced brain injury. We provided an integrated view of NLRP3 regulation in neonatal HIE, with a focus on microglial activation.

### 3.1. NLRP3 Regulation After OGD in Activated Microglia

We established an OGD model in microglial culture to mimic our in vivo hypoxia–ischemia model. Microglia were chosen because they are mediators of inflammatory responses in the brain [15,19,27] and are a key source of NLRP3 in the central nervous system (CNS) [20,28]. Additionally, microglial NLRP3 was previously shown to be increasingly expressed following HI [18,20,29] and inflammation-sensitized HI [18,29].

OGD induced morphological alterations consistent with a reactive microglial phenotype and reduced microglial viability, indicating that OGD elicited a strong microglial response. Our findings suggest a time-dependent involvement of the NLRP3 pathway at the priming stage following OGD, with the greatest transcriptional changes observed at 2 h and 12 h after reoxygenation. Thus, the limited downstream response to OGD likely reflects stimulus- and time-dependent regulation, rather than an inability to detect these components.

In contrast, the secretome analysis at 24 h revealed broader alterations in inflammation-associated proteins. Our observations are consistent with those of previous studies demonstrating NLRP3 pathway activation following OGD in primary microglia and the BV2 microglial cell line [28,30,31].

Particularly, the relevance of the 12 h reoxygenation time point has been reported in BV2 cells [30], HT22 cells [32,33] and primary neurons [6]. Collectively, these findings support the concept that NLRP3 contributes to the early microglial response following OGD, although the precise temporal sequence and downstream mechanisms remain to be elucidated. OGD stimulation of NLRP3 represents stress for the cells, thereby decreasing cell viability. As overactivation of NLRP3 can lead to uncontrolled inflammation and cell death [9,10,11,12], NLRP3 inhibition is thought to be beneficial. The positive effect of NLRP3 inhibition with Crid3 has previously been demonstrated in neurons, endothelial cells and myocytes [32,34,35]. Impaired cell viability due to OGD was rescued [35] and the increased expression of NLRP3 pathway components was prevented [32,34,35].

As NLRP3 is an inflammasome which must be tightly regulated to perform its function in defeating pathogens and infections [9,10,11,12], inhibiting it without another stress stimulus can lead to dysregulated immune defense and thereby activation of microglia. In this study, we showed that NLRP3 inhibition under control conditions led to microglial activation, as quantified by phenotypic analysis, and increased the expression of cleaved caspase-1. Inhibiting NLRP3 without another stimulus can stress cells due to a potential dual role of NLRP3. NLRP3 activity can increase inflammation, but the inflammasome also has repair functions and clears cell debris [36,37]. The detrimental effects of NLRP3 deficiency have been described in previous studies. Mice lacking NLRP3 showed increased brain infarct volume after HI and an increase in activated microglia [38]. Systemic NLRP3 deficiency could potentially be beneficial for continuous NLRP3 overactivation; however, in acute inflammatory disorders, such as HI, acute timed inhibitory treatments may be more effective in limiting inflammatory processes.

Excessive NLRP3 activation by OGD drives uncontrolled inflammation, which can be mitigated by NLRP3 inhibition. However, in the absence of OGD, inhibition of NLRP3 may trigger unintended cell activation. Collectively, these findings indicate that NLRP3 critically regulates microglial activation and dictates their cellular fate.

### 3.2. Microglial NLRP3 Affects Neurons

Several studies have described that microglia influence the fate of neurons [24,25,26,39,40]. Microglia secrete pro- and anti-inflammatory factors which can modulate neuronal functions, and neurons express receptors susceptible to microglia modulation [25,26,41]. We further analyzed microglia–neuron interactions using conditioned medium experiments. OGD-stimulated NLRP3 in microglia leads to increased apoptosis and inflammation in neurons.

While this analysis showed the effect of secreted molecules of microglial cells on neuron-like cells, it did not represent cellular crosstalk. To further study the interactions between cell types, a transwell co-culture system is required, and neurons should be exposed to OGD to study their individual reactions to the stress stimulus. The established neuron-like cell line PC12 was preferred to primary neurons because of its ease of culturing, robust differentiation, and ability to provide higher reproducibility and experimental stability in mechanistic studies [42,43,44].

When analyzing the microglia supernatant using proteomics, seven proteins related to cytokine signaling in the immune system were found to be upregulated due to NLRP3 activation after OGD. Some of these have already been described in the context of HI in previous studies [45,46,47,48,49]. Tollip and Grb2 promote apoptotic processes through Akt and Ras/MAPK (mitogen-activated protein kinase) signaling [47,48,50], and are connected to inflammatory pathways by inducing NF-κB activity [46,47,48]. Grb2 has been described to indirectly promote NLRP3 expression [51]; however, the effect of NLRP3 activation on Grb2 expression has not yet been tested. Tollip has been presented to be negatively associated with NLRP3 expression. Upstream of NLRP3, it acts through the transcription factor NF-κB and suppresses NLRP3 activity [52,53,54]. Nevertheless, the potential feedback loop of NLRP3 activity affecting Tollip expression remains poorly understood and requires further investigation. As both proteins possess pro-apoptotic characteristics by regulating important cellular signaling pathways, these findings validate the results of the present study, showing that microglial NLRP3 promotes the release of molecules leading to apoptosis in neurons.

### 3.3. NLRP3 Inhibition In Vivo

Our results demonstrated a dose-dependent efficiency when inhibiting NLRP3 with Crid3 in the Vannucci neonatal rat model, with the most effective inhibition using 5 mg/kg Crid3 injected 30 min before and 24 h after HI. Although the present data suggest a dose-dependent effect, the limited statistical power precludes definitive conclusions, and experimental heterogeneity cannot be excluded as a contributing factor. Nevertheless, the consistent trend observed across these experiments suggests that a dose of 5 mg/kg produced the strongest inhibitory effect when administered 1 and 24 h after HI. Several previous studies have already presented the effect of Crid3 in adult and neonatal models of ischemic stroke, traumatic brain injury, HI-induced cerebral white matter, or HI brain damage [22,34,55,56] using one concentration of the treatment. Recently, Palomino-Antolin et al. [52] published a study describing Crid3 dose dependency. In an adult mouse model of transient occlusion of the middle cerebral artery (MCAO), the best neuroprotective results were observed when using 3 mg/kg Crid3 1 h after reperfusion [57]. Compared to our model, the animal model (HIE in rats vs. tMCAO in mice) and the age of the animals (neonatal vs. adult) were different. Given the difference between the results, describing the post-HI injection as the most efficient for the tMCAO model [57] and the pre-HI injection for our HIE model, this emphasizes the definite variance between similar but different animal models.

Analysis of the NLRP3 pathway proteins confirmed that 24 h and 48 h after HI were time points of NLRP3 pathway component upregulation. NLRP3 activation at 24 h post-HI or MCAO in neonatal and adult models has been described in several studies. NLRP3 pathway components were found upregulated at mRNA and protein levels [20,21,58]. We observed effective inhibition with Crid3 at both time points. The inhibition with Crid3 was described in several animal models to successfully inhibit NLRP3 activation [22,32,34,35,59,60,61,62,63].

We observed differences in the upregulation of NLRP3 pathway components between the cortex and hippocampus. NLRP3 protein levels were 20 times higher in the cortex, indicating an important role for NLRP3 expression and pathway activation.

Ystgaard et al. [18] reported that the hippocampus had the highest gene expression of NLRP3 when comparing different brain regions [20]. In contrast to previous studies, our findings provide the first protein-level comparison of NLRP3 expression across various brain regions. Protein expression analysis is more reliable than gene expression analysis for determining protein functions. Accordingly, our results indicate a more prominent role for the cortex than the hippocampus in NLRP3 inflammasome activation following experimental HI.

Another difference between the cortex and hippocampus was observed at 24 h, showing that Crid3 treatment significantly inhibited GSDMD-NT protein expression in the hippocampus but not in the cortex. As the last injection of Crid3 was carried out at 24 h and the brains were removed immediately after, the second injection might not have reached the brain tissue. The documented effect at 24 h could be the outcome of the first injection, indicating that the NLRP3 downstream protein GSDMD-NT may require a second injection in the cortex for effective inhibition.

One possible explanation for the hippocampus responding more rapidly than the cortex to the administered treatment is the regional heterogeneity of the blood–brain barrier (BBB). The capillary densities and abundance of cell types differ between brain areas, influencing BBB properties [64]. The hippocampus is more fragile and vulnerable after different treatments, including HI, resulting in a more susceptible BBB [65].

The protein level of NLRP3 increased from 24 h to 48 h, indicating the ongoing activation of the inflammasome. The increase in NLRP3 expression at 24 h to 48 h after HI/Vehicle was already observed by us in isolated microglia [18]. This time span matches the time window of the secondary energy failure following HI [66]. Marked by excitatory, inflammatory, and apoptotic processes, the phase of secondary energy failure aligns with the downstream effects of NLRP3 inflammasome activation, exacerbating brain damage. This highlights the importance of regulating NLRP3 to mitigate brain injury following HI-insult.

### 3.4. Microglial NLRP3 Upregulated After Neonatal HIE

NLRP3 associated genes were upregulated in cortical microglia 24 h after HI. A possible explanation for the gene NLRP3 itself not being upregulated at this time point could be found in our previous articles showing that NLRP3 gene expression was only significantly upregulated when HI was performed with pre-sensitization with LPS [18,29]. We did not find a significant upregulation when HI was performed individually [18,29]. However, analysis of the brain at this time point has already shown HI-induced brain damage in the cortex [67], indicating inflammation. Taken together, NLRP3 gene expression could be upregulated earlier than 24 h, as inflammatory processes were already ongoing, and downstream pathway proteins were upregulated at the transcript level at this time point. This trend of early NLRP3 expression following HI was reported in pilot experiments testing protein levels in brain areas. To fully uncover the role of NLRP3 specifically in microglia in neonatal HIE, an earlier time point analysis needs to be performed in future studies.

Furthermore, analysis at a later time point would be valuable to assess the consequences of NLRP3 inhibition, given that the second treatment injection was administered shortly before sample collection and may require more time to induce changes in gene expression. This could explain why inhibition with Crid3 did not significantly reduce the upregulated gene expression due to HI.

The later time point analysis is consistent with the findings of Ystgaard et al. [20], who reported NLRP3 co-localization with the microglial marker Iba-1 at 72 h after HI. At earlier time points (3 and 24 h), NLRP3 co-localized with astrocytes [20]. The potentially important role of astrocytes in the early regulation of NLRP3 following HI could not be confirmed in the present study, as neither the 3 h time point nor a specific focus on astrocytes was examined. Although not considering other potentially relevant cell populations represents a limitation, focusing on a single cell type enabled the characterization of microglial involvement in HI-induced processes in this study.

### 3.5. Potential Neuroprotective Effect of NLRP3 Inhibition

To link NLRP3 activation and inhibition to long-term effects, we examined brain damage using MRI and conducted behavioral analyses. Seven and 74 days after HI, HI-induced brain damage was reduced by NLRP3 inhibition.

As described in other studies, the inhibition of NLRP3 reduced brain infarct volume in MCAO [32,57], ischemic stroke [34] and hypoxic–ischemic brain damage models [22]. We complemented previous findings by comparing two different time points of analysis in our animal model of HIE and establishing a connection to microglial involvement. Both time points framed the developmental phase of the rats brains, as it takes until three months after birth before the brain is fully developed [68]. During development, HI-induced brain damage did not change with either treatment group. The animal model shows high variability [69,70] with different severities of brain infarcts. The lack of significant neuroprotection may be related to the administration of only two injections at the time of injury. Prolonged systemic treatment may be required to achieve sustained protective effects.

The preservation of cortical MBP immunoreactivity observed following CRID3 treatment is consistent with accumulating evidence that NLRP3 inflammasome activation contributes to white matter injury after neonatal brain injury. In experimental neonatal hypoxic–ischemic white matter injury, pharmacological inhibition of NLRP3 with MCC950 (CRID3) increased MBP expression, preserved myelinated axons, reduced IL-1β production and promoted oligodendrocyte maturation, resulting in improved neurological outcomes [71].

Other recent experimental studies have inhibited NLRP3 activity by upstream targeting of optic atrophy 1 (OPA1) and toll-like receptor 2 (TLR2) and confirmed a connection between the inflammasome and brain damage in neonatal HIE models [23,72].

NLRP3 regulation is critical for neuroprotection. Ystgaard et al. [38] showed that NLRP3 deficiency exacerbated brain injury following HI, highlighting its complex, potentially dual role, whereas ASC deficiency was neuroprotective [38]. While ASC may be a more critical determinant of neuroprotection, NLRP3 remains an important therapeutic target because of its role in regulating ASC activity.

When we analyzed the effect of NLRP3 inhibition in HIE on motor and cognitive skills, NLRP3 showed a trend of rescuing motor impairments induced by HI. Cognitive analysis demonstrated no benefit when inhibiting NLRP3 and no improvement in memory function, distinguishing between new and old objects. The lack of significance in the analysis may represent limited statistical power, as the sample size was insufficient for the high variability of the animal model [69,70]. Recent studies have shown that NLRP3 inhibition improves neurological function in adult MCAO [32,57] and spinal cord injury models [73]. In this study, we linked behavior improvements to our molecular analyses and proposed the involvement of NLRP3. The cortex and hippocampus showed a positive effect on protein expression following HI with NLRP3 inhibition. As the cortex regulates movements [74] and the hippocampus regulates memory functions [75], the effects of NLRP3 inhibition at these early time points could correspond to the long-term outcomes in behavioral assays. In biomolecular analyses, the cortical region presented a higher level of NLRP3 protein than the hippocampus, indicating a more important role for this area in the regulation of inflammasomes. This was confirmed by the behavioral results, which demonstrated that NLRP3 inhibition was only effective in motor, but not in memory functions.

### 3.6. Limitations

Although OGD induced only modest and variable regulation of NLRP3-related genes in isolated primary microglia, the in vivo results provide stronger evidence that the NLRP3 pathway is induced after neonatal HI, while CRID3 attenuated several of these changes, supporting pharmacological regulation of the pathway. The weaker in vitro response should be interpreted in the context of this cell-specific experimental model, which lacks multicellular interactions, circulating immune cells, DAMP release, and sustained inflammatory signaling present in the injured brain. Moreover, the in vitro analysis captured selected transcriptional time points, whereas inflammasome regulation is highly dynamic and occurs substantially at the level of protein assembly, cleavage, and oligomerization. Thus, the limited transcriptional response observed in vitro likely reflects the experimental conditions and temporal sampling rather than the absence of NLRP3 involvement. Collectively, the in vitro findings provide mechanistic support, whereas the more physiologically relevant in vivo data demonstrate the regulation of the NLRP3 after neonatal HI.

For a better comparison between molecular analyses and behavior assays, protein expression analysis should be repeated at the same time point when long-term effects are tested. As the brain tissue of rats at this late time point is too damaged to distinguish between different brain areas, this limitation rules out the potential for this analysis.

Microglia were isolated following HI at the time point of 24 h because this time point showed pathway activation in previous experiments. Nonetheless, analyzing a single time point limits the ability to fully uncover the role of time-critical microglial NLRP3 activation. Other time point analyses should be addressed in future studies.

We used the NLRP3 inhibitor, Crid3. This inhibitor has already been tested in a phase II clinical trial and was documented to induce liver toxicity [76]. We are aware that our treatment will not be translational in clinical studies. However, as we used this inhibitor only for mechanistic studies, we relied on it because of its high specificity [77]. Alternative NLRP3 inhibitors have also been widely studied. Their mechanisms of action are often unknown, and their potency is lower than that of Crid3. Nonetheless, promising small-molecule inhibitors, such as the potent primate-NLRP3-inhibiting BAL-0028, have been discovered by Wilhelmsen et al. [78] and the selective NLRP3 inhibitor OLT1177 (dapansutrile), which has been evaluated in phase II clinical trials and shown to have anti-inflammatory effects [79,80]. Future studies should address the use of alternative inhibitors to improve the translational applicability of this study.

The use of Crid3 as a single-agent pharmacological inhibitor of NLRP3 requires validation by genetically knocking out NLRP3 or using alternative drugs that inhibit NLRP3. As systemic genetic knockout of NLRP3 has already been shown to worsen brain damage in a mouse HI model [38], acute treatments with inhibitory drugs are more promising for providing effective treatments. However, the lack of validation of NLRP3 specificity represents a major limitation of this study.

The present study is limited by its statistical power, which restricts the strength of the conclusions. Although some findings did not reach statistical significance, similar trends were observed across independent experiments, suggesting that the overall pattern of the results is reproducible. Future studies with larger sample sizes will be necessary to validate these findings, further strengthen the statistical evidence, and improve the power of subgroup analyses, particularly in vivo data assessing sex-specific effects.

Additionally, the statistical approach involving multiple pairwise comparisons may have reduced the power to detect more subtle differences between individual groups. While this approach allowed for a comprehensive assessment of potential differences across treatment groups, future studies may provide increased statistical power using pre-specified comparisons.

In this study, we present the first results of an investigation into the effect of NLRP3 inhibition in our animal model. Alternative validation methods were not included in the current study but will be addressed in future studies.

## 4. Materials and Methods

### 4.1. Animal Experiments

Animal experiments using Wistar rats (Janvier Labs, Le Genest-Saint-Isle, France) were approved by the North Rhine-Westphalia State Office for Consumer Protection and Nutrition (LAVE, Recklinghausen, Germany) and were conducted according to the ARRIVE guidelines. Animals were kept at the German Center for Neurodegenerative Diseases (DZNE) Bonn, Germany, under a 12:12 h light/dark cycle at 21 °C with food and water provided ad libitum.

HI was performed using the Vannucci model, as previously described [81,82]. Briefly, postnatal day 7 Wistar rats were randomized according to litter, sex, and weight. Following buprenorphine analgesia (Bupaq, VetVia Richter GmbH, Wels, Austria), the animals were anesthetized with 5% isoflurane (Virbac, Carros, France), the left common carotid artery was ligated, and the animals were exposed to hypoxia (8% O_2_) for 90 min. The core temperature (T_rec_; IT-21, Physitemp Instruments, LLC, Clifton, NJ, USA) was maintained at 36 °C using a servo-controlled cooling mat (water pad, Hirtz & Co. KG, Cologne, Germany), as previously described [18,83]. After 90 min, T_rec_ was set to 37 °C, and the animals were maintained under normoxia (21% O_2_) for 5 h before being returned to their dam. Sham animals received 5 min anesthesia (5% isoflurane) without ligation.

We used pharmacological NLRP3 inhibition as a mechanistic tool to dissect the inflammasome-dependent pathways following neonatal hypoxia–ischemia. To determine the therapeutic window and dose–response of the treatment, animals were subjected to different concentrations of the drug at different time points. These were determined after the first pilot experiments testing different concentrations and injection time points, following research on similar experimental models (Supplementary Table S1) [22,55,57,84,85]. The following treatment groups were selected to establish injection conditions for NLRP3 inhibition with Cytokine Release Inhibitory Drug 3 (Crid3; also known as MCC950): (1) 5 mg/kg Crid3; (2) 10 mg/kg Crid3; (3) 20 mg/kg Crid3 (5.38120, Sigma-Aldrich, St. Louis, MO, USA). Groups 1–3 were injected either 30 min before and 24 h after HI or 1 h after and 24 h after HI, resulting in six different groups (Supplementary Figure S1). All treatments were administered intraperitoneally (i.p.) (0.1 mL/10 g body weight). Simultaneously, a vehicle solution (NaCl 0.9%) was injected as a control. Treatment with Crid3 did not increase the death rate (survival rate in Supplementary Table S2). A total of 178 animals were used in this study (Supplementary Table S2).

### 4.2. Western Blot

Brain tissue or cells were lysed as previously described [82] and the protein concentration was quantified using a BCA assay (Pierce™ BCA Protein Assay Kits, Thermo Scientific™, Life Technologies Corporation, Carlsbad, CA, USA). Brain tissue (50 µg protein) or cells (20 µg protein) were loaded onto SDS-PAGE (SDS-pellets, Carl Roth, Karlsruhe, Germany) and transferred to a polyvinylidene difluoride membrane. After blocking the membrane with 5% *w*/*v* milk, the primary antibody was incubated overnight at 4 °C (Supplementary Table S3). After washing, the secondary antibody was incubated for 1 h (Supplementary Table S3). Proteins were visualized using an iBright 1500 system (Invitrogen, Life Technologies Corporation, Carlsbad, CA, USA). The expression levels of individual proteins were normalized to those of β-actin.

### 4.3. Magnetic-Activated Cell Sorting (MACS)

At 24 h after HI, magnetic-activated cell sorting was performed to isolate microglial cells from the ipsilateral cortex, as previously described [18]. Brain tissue was collected in HBSS and homogenized using a 40 µm cell strainer. After centrifugation, the cell pellet was resuspended in myelin removal beads (Miltenyi Biotech, Bergisch Gladbach, Germany) for 15 min and washed. LS columns were used to clean the myelin sample. The sorted cells were incubated with CD11b/c beads (Miltenyi Biotech, Bergisch Gladbach, Germany) for 15 min and then washed. MS columns were used to collect the microglia. Sorted microglia were counted and analyzed via fluorescence-activated cell sorting (FACS) and RT-PCR.

### 4.4. Fluorescence-Activated Cell Sorting (FACS)

Microglia (1.9 × 10^4^) were pipetted into a 96 well plate and centrifuged. The supernatant was discarded, and the cells were incubated with an antibody mix for 30 min on ice (Supplementary Table S4). After washing, the cells were resuspended in PBS and analyzed using FACS (BD FACS SymphonyTM A5, BD Biosciences, San Jose, CA, USA). Gating was performed as follows: 1- FSC-A vs. SSC-A, 2- singlets FSC-H vs. FSC-A, 3- Live cells, using zombie-UV (BD Bisociences, San Jose, CA, USA), 4- Leucocytes vs. Myeloid cells using CD45 Brilliant Violet 711 (BV711) (leucocyte common antigen, microglia marker), and CD11b/c Phycoerythrin (PE) (microglia marker). Microglia cells were considered CD45^high^ CD11b^+^ (Figure 8). A total of 50,000 events were recorded and analyzed using FlowJo software version 11 (BD Biosciences, San Jose, CA, USA). Purity over 90% was considered for further studies.

### 4.5. RT-PCR

Microglial RNA was extracted using TRIzol (Life Technologies Corporation, Carlsbad, CA, USA) and chloroform (Merck Millipore, Burlington, MA, USA). cDNA synthesis was performed using a high-capacity cDNA reverse transcription kit (4368814, Thermo Fisher Scientific, Life Technologies Corporation, Carlsbad, CA, USA). The PCR reaction was carried out under the following conditions: 95 °C, 20 s, denaturation; 95 °C, 1 s; 60 °C, 20 s; and 40 cycles. The primers used are listed in Supplementary Table S5. Results are presented using the 2^–∆∆Ct^ method (Normalization to β-actin and the corresponding control condition).

### 4.6. Magnetic Resonance Imaging (MRI)

MRI was performed at 7 and 74 days after HI, as previously described [82]. The animals were anesthetized with 5% isoflurane. An 11.7 T horizontal small-bore magnet (Biospec 117/16; Bruker, Billerica, MA, USA) and a rat brain receive only proton (1H) coil (Bruker Biospin) were used to scan the brains of the animals from anterior to posterior in the sagittal direction, with a focus on the Bregma region 0.2 to −7 mm. A rapid acquisition relaxation enhancement T2-weighted sequence with an echo time of 25 ms, repetition time of 2.9 s, and in-plane resolution of 0.156 × 0.156 mm^2^ was used for image acquisition. Images were analyzed by measuring the edema size using ImageJ (Fiji, ImageJ, version 1.54p)on the Bregma regions −0.88 mm and −2.8 mm, which represent the vulnerable brain areas in this model. The areas of the ipsilateral and contralateral brains and edema were measured. The percentage area loss was calculated using the formula: 100 × (1 − ((brain ipsilateral − edema ipsilateral)/brain contralateral)) in both regions, and the average was calculated.

### 4.7. Catwalk Test

Behavioral testing using Catwalk was performed 46–48 days after HI, as previously described [82]. The animals were recorded using a camera from below while walking through a passage on an illuminated glass plate. During the two days of training, the animals performed three runs in a maximum of 5 s each. On the third day, the animals were subjected to the same tasks. The average of the three runs was analyzed using the Catwalk XT 10.6 software (Noldus Information Technology, Wageningen, The Netherlands).

### 4.8. Novel Object Recognition Test

Cognitive behavior was tested using novel object recognition (NOR) at 74–76 days after HI, as previously described [82]. The animals were recorded with a camera from above for 5 min when placed in a 48 × 48 cm box and exposed to different objects. During the two days of training, the animals were habituated to two identical objects placed in the opposite corners of the box. On the third day, the animals were again exposed to the same objects for 5 min, followed by a break of 1 h, after which they were exposed to a new object. The analysis was performed using the EthoVision XT 17.5 analysis system (Noldus Information Technology, Wageningen, The Netherlands).

### 4.9. Culture of Primary Rat Microglia and Oxygen–Glucose Deprivation (OGD)

Primary microglia were isolated from P1 Wistar rats. Brain tissue (excluding the cerebellum) was separated from the meninges and mechanically dissociated in cold HBSS. After 5 min of digestion with 0.25% *v*/*v* trypsin/EDTA (gibco, Life Technologies Corporation, Carlsbad, CA, USA), the suspension was passed through a 40 µm cell strainer and centrifuged. Cells were plated on poly L-lysine-coated T75 culture flasks (two brains per flask) in DMEM (10% *v*/*v* FCS, 1% *v*/*v* penicilin/streptomycin, PAN-Biotech GmbH, Aidenbach, Germany). The next day, the medium was replaced with DMEM containing 20 ng/mL GM-CSF (R&D Systems, 518-GM). The effect of GM-CSF was assessed in pilot experiments by testing cell death and proliferation via cell counting. After 7 days, microglia were shaken off the astrocyte layer and plated on coated plates at a density of 0.3 × 10^6^ cells/mL. A purity threshold of 90% was applied. The cells were subjected to oxygen–glucose deprivation (OGD) the following day.

OGD was performed in a hypoxic chamber (0.1% O_2_, 5% CO_2_, 37 °C; InvivO_2_ 400, Baker Ruskinn, Sanford, ME, USA). Cells were washed and incubated in glucose-free DMEM for 6 h (pilot experiments; cell death < 30%). The medium was then replaced with culture medium and the cells were returned to normoxia. Control cells were maintained under standard culture conditions.

NLRP3 inhibition was achieved by incubating the cells with 1 µM Crid3 (Tocris) for 30 min before and during OGD. Lipopolysaccharide (LPS) served as a positive control (100 ng/mL, 6 h; Sigma-Aldrich, St. Louis, MO, USA).

### 4.10. PC12 Cell Culture

To assess the effects of activated microglia on neuronal cells, PC12 were used (Cat. N: CLS-500311, Hölzel, Cologne, Germany). The PC12 cells were obtained from Hölzel-Biotech (Cat. N: CLS-500311). PC12 cells derived from a rat adrenal gland tumor (pheochromocytoma) and are used in our research because they can develop neuron-like characteristics and extend neurites when treated with nerve growth factor (NGF) [43]. Cells were cultured in coated T75 flasks in RPMI medium (10% HS, 5% FBS, 1% Pen/Strep). For differentiation, cells were plated at 100,000 cells/mL in medium supplemented with 1% *v*/*v* non-essential amino acids, 1% *v*/*v* sodium pyruvate, and recombinant rat β-NGF (100 ng/mL; R&D Systems, 556-NG). After 2 days, NGF was removed by changing the medium, and the cells were used for experiments after 2 days.

Microglia supernatant was collected 24 h after OGD and added to PC12 cells for 24 h at a 1:1 ratio with PC12 culture medium. The controls included PC12 medium alone and a 1:1 mixture of PC12 and microglial culture medium. After incubation, the cells were collected for Western blotting or cell viability analysis.

### 4.11. Cell Viability Assay

For the cell viability assay, an MTT assay kit (ab211091-Abcam, Cambridge, UK) to quantify viable cells. The cells were incubated with MTT reagent for 4 h at 37 °C in the dark. MTT was converted by viable cells into formazan, which was measured by absorbance at 590 nm. DMSO (AppliChem GmbH, Darmstadt, Germany) was added and mixed, before the absorbance was measured at 590 nm wavelength. Wells without plated cells were used for background subtraction.

### 4.12. ELISA

The supernatant of microglial cells 24 h after OGD was analyzed using an enzyme-linked immunosorbent assay (ELISA) to quantify the released IL-1β. Prior to the ELISA (KE20021, Proteintech Group, Rosemont, IL, USA), the supernatant was enriched using an Amicon^®^ Ultra Centrifugal Filter (UFC201024, Merck Millipore, Burlington, MA, USA), as described by the manufacturer. Briefly, 1 mL of the sample was added to a centrifugal filter device, and the volume was reduced to 200 µL by centrifugation.

### 4.13. Immunofluorescence

The cells were washed and fixed in cold 100% *v*/*v* methanol. Blocking was conducted with 20% *v*/*v* goat serum for 30 min, and the primary antibody was incubated overnight at 4 °C (anti-Iba-1/019–19741/Wako/1:400, FUJIFILM Wako Chemicals Europe GmbH, Neuss, Germany), followed by the secondary antibody for 1 h (Goat anti-rabbit 488/11008/Invitrogen/1:500, Life Technologies Corporation, Carlsbad, CA, USA). DAPI was stained (Fluoromount G™ Mounting Medium with DAPI, Invitrogen, Life Technologies Corporation, Carlsbad, CA, USA). After imaging with a confocal microscope (LSM800, Carl Zeiss Microscopy Deutschland GmbH, Oberkochen, Germany), analysis was performed using the ImageJ (Fiji, ImageJ, version 1.54p) plugin “NeuronJ” to measure the process length and number of processes per cell.

### 4.14. Proteomic Analysis of Microglia Supernatant via Mass Spectrometry

Liquid chromatography–mass spectrometry/mass spectrometry (LC-MS/MS) was used to analyze newly synthesized and released proteins in microglia subjected to different treatments. Microglia were depleted of methionine for 30 min and incubated with 2 mM l-azidohomoalanine (AHA, Iris Biotech GmbH, Marktredwitz, Germany) to label newly synthesized proteins. After 24 h, the cell supernatants were collected.

Samples were processed using a click-chemistry-based enrichment workflow to isolate newly synthesized proteins, followed by on-bead digestion and data-independent acquisition (DIA)-based proteomic analysis. DIA data were processed using DIA-NN in library-free mode with in silico spectral prediction and two-pass library refinement, followed by downstream statistical analysis in R using MSstats and limma (R Foundation for Statistical Computing, Version 4.5.1, Vienna, Austria). All detailed experimental parameters, reagent compositions, instrument settings, and data-processing steps are provided in the (Supplementary Material S6).

Normalization per condition was used for the evaluation. The effect of NLRP3 activation and inhibition was investigated by including log^2^ fold change (log2FC) values with a minimum of 2 for the comparison of OGD vs. CTR and OGD vs. OC and with a maximum of 1.5 for the comparison of OC vs. CTR. The STRING database (string-db.org) was used for the analysis.

### 4.15. Immunofluorescent Staining

Brain sections were deparaffinized in xylene, followed by rehydration in decrease-ethanol concentrations. For antigen retrieval, sections were heated to 95 °C in 10 mM sodium citrate buffer (pH 6.0) for 10 min. Samples were permeabilized with 0.1% Triton X-100 (Sigma-Aldrich, St. Louis, MO, USA) in 1× PBS for 10 min and blocked with 20% *v*/*v* goat serum for 1 h at room temperature, followed by overnight incubation with primary antibodies against MBP (1:500, Cell Signaling Technology, 78896, Inc., Danvers, MA, USA) diluted in PBS. The next day, sections were washed three-times in PBS and incubated with Alexa-fluor conjugated secondary antibodies goat-anti-rabbit AF488 (1:500, Life Technologies Corporation, Carlsbad, CA, USA) for 1 h at room temperature. The sections were embedded using FluorSave with DAPI. Fluorescent staining was visualized using Axioscan Z1 (Carl Zeiss Microscopy Deutschland GmbH, Oberkochen, Germany). To measure the thickness of myelinated cortex, one picture of each hemisphere in the rat model was acquired at 2.5× magnification, as previously described [86].

The thickness of myelinated cortex was assessed by measuring the area of the cortical tissue above the external capsule on 2.5× images and by measuring the area of myelinated cortex. The proportion of myelinated cortex was calculated as (myelinated cortex/total cortex) × 100% [86].

### 4.16. Statistical Analysis

Statistical analyses were performed using GraphPad Prism version 10.4.2 software (GraphPad Software, San Diego, CA, USA). Biological replicates (BR; individual animals for in vivo experiments and independent cell cultures for in vitro experiments) were considered experimental units. Technical replicates (TR; e.g., wells) were averaged prior to statistical analysis to avoid pseudoreplication. Normality was assessed using the Shapiro–Wilk test, and non-parametric tests were applied throughout. For comparisons involving more than two groups, the Kruskal–Wallis test was used, followed by Dunn’s multiple comparisons test with adjustment for multiple comparisons, as implemented in GraphPad Prism software. Dunn’s post hoc test was applied for pairwise comparisons between all groups within each analysis. A significance level of α = 0.05 was used (* *p* ≤ 0.05; ** *p* ≤ 0.01; *** *p* ≤ 0.001). All tests were two-tailed, and exact *p*-values (adjusted for multiple comparisons where applicable) are reported in the figures, with relevant test statistics provided in the figure legends (Kruskal–Wallis statistic *H*). Data are presented as median ± interquartile range (IQR). The exact *N* values are reported in the figure legend.

## 5. Conclusions

In conclusion, our study demonstrates that neonatal HI is associated with the activation of NLRP3-related signaling both in vitro and in vivo, and that pharmacological inhibition with CRID3 modulates several molecular, histological, and functional outcomes after injury. By integrating complementary experimental approaches, we provided evidence supporting the involvement of NLRP3 in the inflammatory response following neonatal HIE, with particular emphasis on microglial cells. At the same time, our findings highlight the complexity of NLRP3 signaling and indicate that its temporal and cell-specific regulation requires further investigation. These findings represent the first step towards identifying microglial NLRP3 as a potential therapeutic target for HIE.

## Data Availability

The data that support the findings of this study are available from the corresponding author upon reasonable request.

## Supplementary Materials

The following supporting information can be downloaded at: https://www.mdpi.com/article/10.3390/ijms27177853/s1.

## Abbreviations

Hypoxic–ischaemic encephalopathy	HIE
Hypoxic–ischaemic	HI
Therapeutic hypothermia	TH
NLR family pyrin domain containing 3	NLRP3
Damage- associated molecular patterns	DAMPs
Pathogen- associated molecular patterns	PAMPs
Apoptosis-associated speck-like protein containing a CARD	ASC
Pore-forming protein Gasdermin D	GSDMD
Oxygen–Glucose Deprivation	OGD
Cytokine Release Inhibitory Drug 3	Crid3
Lipopolysaccharide	LPS
B-cell lymphoma 2	BCL-2
BCL-2 associated X protein	BAX
