# Supplementary material for: NLRP3 Regulation in Neonatal Hypoxic–Ischemic Encephalopathy—Focus on Microglial Activation
Source: Int J Mol Sci. 2026 Sep 2;27(17):7853. doi: 10.3390/ijms27177853 (PMC13566588; doi:10.3390/ijms27177853)

Supplementary Material S1

Supplementary Figure S1 Experimental timeline describing injection time points of Crid3 treatment. Wistar rats at P7 undergo left common carotid artery ligation and hypoxia for 90 min. Rats get i.p. injected at 30 min before and 24 h after HI (injection scheme 1) or at 1 h after and 24 h after HI (injection scheme 2) with 5/10/20 mg/kg Crid3 or vehicle (saline). Brain areas were taken at 48 h after HI and analyzed via western blot.

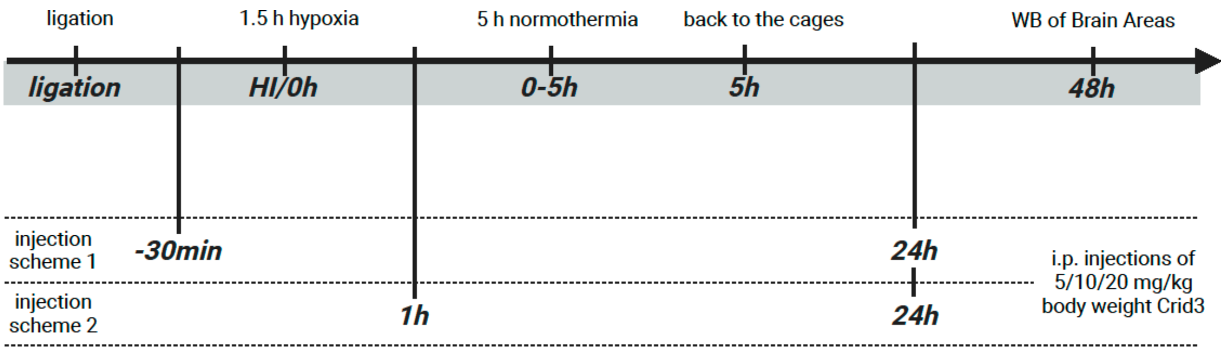

Supplementary Material S2

Supplementary Table S1 Overview of pilot experiments treating neonatal hypoxic-ischemic encephalopathy with the NLRP3 inhibitor Crid3. Listed details about concentration and injection time points of Crid3.

Experiments	Concentration	Injection time points						Outcome/ inhibition
		before HI	after HI					
1st experiment	50 mg/kg		0 h	4 h		24 h		not effective
2nd experiment	25 mg/kg		0 h	3 h	6 h	24 h		
3rd experiment	20 mg/kg	30 min	0 h		6 h	24 h		
4th experiment	3 mg/kg			1h				
	5 mg/kg			1h				
5th experiment	10 mg/kg	30 min				24 h	48 h	

Supplementary Material S3

Supplementary Table S2 List of animals used for experiments.

Experiment	Groups	Time points for		Analysis method	Animals		
		Injection	Analysis		Analyzed	♂ / ♀	Died
Hypoxia-Ischemia/ Inhibitor treatment	Sham		24 h	WB	5	2/3	-
	HI/Vehicle	- 30 min/ + 24 h	24 h	WB	16	8/8	-
	HI/Crid		24 h	WB	16	8/8	-
	Sham		24 h	MACS	6	3/3	-
	HI/Vehicle	- 30 min/ + 24 h	24 h	MACS	12	6/6	-
	HI/Crid		24 h	MACS	12	6/6	-
	Sham		48 h	WB	5	3/2	-
	HI/Vehicle	- 30 min/ + 24 h	48 h	WB	9	5/4	3
	HI/Crid5		48 h	WB	7	3/4	3
	HI/Crid10		48 h	WB	7	5/2	3
	HI/Crid20		48 h	WB	6	4/2	4
	HI/Vehicle	+ 1 h/ + 24 h	48 h	WB	8	4/4	1
	HI/Crid5		48 h	WB	9	6/3	-
	HI/Crid10		48 h	WB	8	5/3	-
	HI/Crid20		48 h	WB	8	6/2	-
	Sham		P 14	MRI	5*	2/3	-
	HI/Vehicle	- 30 min/ + 24 h	P 14	MRI	12*	7/5	-
	HI/Crid		P 14	MRI	12*	7/5	-
	Sham		P 46-48	Catwalk	5*	2/3	-
	HI/Vehicle	- 30 min/ + 24 h	P 46-48	Catwalk	12*	7/5	-
	HI/Crid		P 46-48	Catwalk	12*	7/5	-
	Sham		P 74-76	NOR	5*	2/3	-
	HI/Vehicle	- 30 min/ + 24 h	P 74-76	NOR	12*	7/5	-
	HI/Crid		P 74-76	NOR	12*	7/5	-
	Sham		P 81	MRI	5*	2/3	-
	HI/Vehicle	- 30 min/ + 24 h	P 81	MRI	12*	7/5	-
	HI/Crid		P 81	MRI	12*	7/5	-

Supplementary Material S4
Supplementary Table S3 Antibodies used for western blot.

Primary antibody	Company	Catalogue number	Dilution
ASC	Novus Biologicals, Centennial, CO, USA	NBP1-78977	1:250
β-Actin	Sigma-Aldrich, St. Louis, MO, USA	A1978	1:3000
BAX	abcam, Cambridge, UK	ab32503	1:1000
BCL-2	abcam, Cambridge, UK	ab196495	1:1000
Caspase-1	Affinity Biosciences, Cincinnati, OH, USA	AF5418	1:1000
Caspase-9	Proteintech Group, Rosemont, IL, USA	10380-1-AP	1:500
Cleaved Caspase-1	Cell Signaling Technology, Danvers, MA, USA	89332	1:250
GSDMD	abcam, Cambridge, UK	ab219800	1:500
IL-1b	abcam, Cambridge, UK	ab9787	1:200
IL-18	abcam, Cambridge, UK	ab191860	1:250
NLRP3	abcam, Cambridge, UK	ab263899	1:1000
**Seccondary antibody**	**Company**	**Catalogue number**	**Dilution**
Goat Anti-Rabbit	Jackson ImmunoResearch, West Grove, PA, USA	111-035-003	1:10,000
IRDye 680RD Goat Anti-Mouse	LI-COR, Lincoln, NE, USA	926-68070	1:3000
IRDye 800CW Goat anti-Rabbit	LI-COR, Lincoln, NE, USA	926-32211	1:5000

Supplementary Material S5

Supplementary Table S4 List of antibodies used for FACS.

Antibody	Company	Ca. No.	Dilution
PE anti-rat CD11b/c Antibody	BioLegend, San Diego, CA, USA	201807	1:100
BD Pharmingen™ Purified Mouse Anti-Rat CD32	BD Biosciences, San Jose, CA, USA	550270	1:100
BD OptiBuild™ BV711 Mouse Anti-Rat CD45	BD Biosciences, San Jose, CA, USA	740725	1:100
Zombie UV™ Dye	BioLegend, San Diego, CA, USA	77474	1:100

Supplementary Material S6

Supplementary Table S5 Primers used for RT-PCR.

Primer target	Assay ID	Company
ASC	Rn00597229_g1	Thermo Fisher Scientific, Waltham, MA, USA
β-Actin	Rn00667869_m1	Thermo Fisher Scientific, Waltham, MA, USA
Caspase-1	Rn00562724m1	Thermo Fisher Scientific, Waltham, MA, USA
GSDMD	Rn06429108_s1	Thermo Fisher Scientific, Waltham, MA, USA
IL-1b	Rn00580432m1	Thermo Fisher Scientific, Waltham, MA, USA
NLRP3	Rn04244620m1	Thermo Fisher Scientific, Waltham, MA, USA

Supplementary Material S7

Detailed Proteomics procedure

Preparation of Alkyne-Functionalized Magnetic Beads

Epoxy-activated magnetic agarose beads (Cube Biotech, 50805, Monheim am Rhein, Germany) were functionalized with propargylamine following the protocol described by Bortecen and collegues (doi:10.1038/s41467-023-43919-3). Briefly, beads were washed and incubated in coupling solution containing propargylamine (Santa Cruz Biotechnology, sc-253321A, Dallas, TX, USA) dissolved in dipotassium phosphate (Carl Roth, P749.1, Karlsruhe, Germany) for 16 h at 45 °C with shaking. After washing the beads, they were incubated in quenching buffer for 4 h at 45 °C, washed again and stored at 4 °C in storage buffer.

Sample Preparation, Lysis, and Protein Quantification

Thawed cell culture supernatants were filtered with a 3 kD Merck Amicon® filters (Merck Millipore, UFC200324, Burlington, MA, USA). The remaining material (approx. 100 µl) was sonicated on ice (3 cycles, 5 s on/5 s off, 30% amplitude) in 1 ml of freshly prepared guanidinium chloride (GdmCl) lysis buffer (6 M GdmCl (Merck Millipore, G3272, Burlington, MA, USA), 200 mM HEPES pH 8.0 (Fisher, 15630080, Thermo Fisher Scientific, Waltham, MA, USA), 4% CHAPS (Sigma-Aldrich, 220201, St. Louis, MO, USA), and 0.5 M NaCl (Sigma-Aldrich, S9888, St. Louis, MO, USA), supplemented immediately before use with protease inhibitor (PI) cocktail (Roche cOmplete™ , 11836170001, Roche Diagnostics GmbH, Mannheim, Germany)). Protein concentrations were measured using the Pierce BCA Protein Assay Kit (Thermo Fisher Scientific, 23225, Waltham, MA, USA) according to the manufacturer's protocol. Samples and standards were run in duplicate, and all lysates were normalized to equal protein input for downstream copper-catalyzed azide-alkyne cycloaddition (CuAAC) enrichment.

Click-Chemistry Enrichment of Newly Synthesized Proteins

Functionalized magnetic beads were washed twice in GdmCl lysis buffer and resuspended in GdmCl-PI buffer. For each reaction, 10 µl bead slurry was added. For each 1 ml reaction, the following were combined in order: 835 µl ultrapure water, 125 µl 100 mM BTTAA ligand (Lumiprobe, 3253,

Hannover, Germany), 20 µl 100 mM $CuSO_4$ solution (Sigma-Aldrich, C1297, St. Louis, MO, USA), 20 µl 1 M ascorbic acid (Sigma-Aldrich, A92902, St. Louis, MO, USA). Bead-lysate mixtures were adjusted to a total reaction volume of 2 ml and incubated overnight at RT while rotating at 8 rpm.

Detergent Removal, Reduction, Alkylation, and On-Bead Digestion

Following CuAAC click reaction, samples were pelleted (600 × g, 30 s) and placed on a magnetic rack and supernatants were discarded. Beads were washed with SDS wash buffer (100 mM Tris-HCl pH 8.0 (Thermo Fisher Scientific, 15568025, Waltham, MA, USA), 1% SDS (Sigma-Aldrich, L3771, St. Louis, MO, USA), 250 mM NaCl, and 5 mM EDTA (Sigma-Aldrich, E9884, St. Louis, MO, USA)). After pelleting (600 × g, 30 s), beads were resuspended in 500 µl freshly prepared reduction/alkylation buffer (10 mM tris(2-carboxyethyl)phosphine (TCEP; Thermo Fisher Scientific, 77720, Waltham, MA, USA) and 40 mM 2-chloroacetamide (CAA; Sigma-Aldrich, C0267, St. Louis, MO, USA) in SDS). Samples were incubated for 15 min at 70 °C (800 rpm). Beads were washed thrice with SDS buffer, twice with 70% ethanol (Merck Millipore, 100983, Burlington, MA, USA), and once with 100% LC-MS-grade acetonitrile (Thermo Fisher Scientific, A955-4, Waltham, MA, USA). Proteins were digested on-bead by resuspending beads in 100 µl digestion buffer (100 mM Tris-HCl pH 8.0, supplemented with Trypsin/Lys-C Mix (Promega, V5073, Madison, WI, USA) at 1 µl per reaction) and an overnight incubation at 37 °C at 800 rpm.

Peptide Cleanup Using SDB-RPS StageTips

Peptide digests were acidified to approximately 1% formic acid (Merck Millipore, 5330020050, Burlington, MA, USA). Two-disk SDB-RPS StageTips were assembled using CDS Empore™ SDB-RPS Extraction Disks (SPE-Disks-Bio-RPS-M-47.20). StageTips were activated (100% methanol), washed with Buffer B (80% acetonitrile, 0.1% formic acid), and equilibrated twice with 100 µl Buffer A (0.1% formic acid in LC-MS grade water). Up to 40 µg peptides (20 µg per disk) were loaded by centrifugation. StageTips were washed with Buffer A, followed by two washes of Buffer B. Peptides were eluted using 60 µl freshly prepared basic elution buffer (80% acetonitrile, 5% ammonium hydroxide solution; 25% NH_3, Carl Roth 6774, Karlsruhe, Germany), dried in a vacuum concentrator, and resuspended in 12 µl Buffer R (2% acetonitrile, 0.1% formic acid). Peptide

concentrations were determined on a NanoDrop OneC (Thermo Fisher Scientific, Waltham, MA, USA), and 300 ng peptide was injected for LC-MS/MS.

LC-MS/MS Acquisitions

Peptides were analyzed on a Thermo Scientific Orbitrap Exploris 480 mass spectrometer coupled to a Thermo Scientific Vanquish Neo UHPLC operated in trap-and-elute mode. Instrument control was performed using Xcalibur v4.7.69.37 (Thermo Fisher Scientific, Waltham, MA, USA) and Tune v4.2.362.26 (Thermo Fisher Scientific Inc., Waltham, MA, USA). Samples were loaded onto a 300 µm × 5 mm trap column and separated on a 75 µm × 25 cm analytical column at a flow rate of 0.35 µl/min. Mobile phase A consisted of water with 0.1% formic acid, and mobile phase B consisted of 80% acetonitrile with 0.1% formic acid. The 120-min gradient increased from 2% to 6% B (1 min), from 6% to 30% B (1-96 min), then to 55% B at 109 min, followed by a wash at 95% B until 120 min. The autosampler was maintained at 7 °C. MS1 scans were acquired in the Orbitrap from m/z 300-1650 at 120,000 resolution (at m/z 200) with a maximum injection time of 60 ms and an AGC target of 300%. One microscan was used, and data were collected in profile mode. The spray voltage was 2.4 kV and the ion transfer tube temperature was 250 °C. DIA MS2 spectra were acquired using tMS^2 with 32 variable isolation windows (window widths ~16-93 m/z).

DIA label-free quantification

For the analysis of DIA raw data, we used DIA-NN (version 2.2.0) in library-free mode with in-silico prediction and the --export-quant option enabled. A spectral library was first predicted from the FASTA database (Rattus norvegicus, UniProt SwissProt, downloaded May 2023), using an in-silico digest allowing tryptic cleavage after K/R, up to two missed cleavages, peptide lengths between 7-30 amino acids, precursor charge states +1 to +4, and m/z ranges of 300-1000 (precursor) and 100-2000 (fragment ions). The following modifications were specified: carbamidomethylation of cysteine (fixed), N-terminal methionine excision, methionine oxidation, and N-terminal acetylation as variable modifications, with a maximum of three variable modifications per peptide. DIA-NN was configured to use isotope detection, peptidoform scoring, heuristic protein inference, and exclusion of shared

spectra, with match-between-runs (MBR) enabled. Mass accuracy parameters were set to 30 ppm for MS2 and 15 ppm for MS1, applied uniformly across all runs, with run-specific individual mass-accuracy calibration (--individual-mass-acc) and window inference (--individual-windows). DIA-NN was run with RT-dependent cross-run normalization, robust LC quantification, smart profiling library generation, and the single-pass neural network classifier. All files were first processed to generate an empirically refined DIA-based spectral library (first-pass), which was subsequently used in a second-pass re-analysis of all raw files to increase identification accuracy and retention-time alignment. Full precursor- and protein-level quantification matrices were exported using the --matrices and --export-quant commands. All analyses were controlled at 1% precursor- and protein-group FDR using DIA-NN's built-in q-value estimation. Resulting *report.parquet* served as the basis for all downstream processing.

Data processing

DIA-NN output files (*report.parquet* and quantification matrices) were processed in R (v4.5.1; R Foundation for Statistical Computing, Vienna, Austria) via RStudio (v2025.05.1; Posit Software, PBC, Boston, MA, USA) using MSstats (v4.16.1) and limma (v3.64.1). DIA-NN tables were converted using DIANNtoMSstatsFormat (useUniquePeptide=TRUE, useZero Intensity=FALSE, summaryMethod="TMP", removeProtein_with1Feature =TRUE, equalFeatureVariance=TRUE), followed by log2 transformation. Because non-clicked control samples contain substantially fewer enriched peptides, we applied a condition-based median normalization to adjust for this systematic difference in total signal, ensuring comparability while preserving within-condition structure. Protein-level abundances were estimated using MSstats::dataProcess (TMP summarization; normalization ="none"; censoring enabled but without MSstats internal MBimpute). Remaining missing values in the summarized protein matrix were then imputed post-MSstats using a left-censored approach: values were drawn from a downshifted (~1.8 SD) and narrowed (~0.3 SD) normal distribution within each condition to approximate low-abundance signals. Differential protein abundance was assessed using limma, fitting linear models with empirical Bayes moderation and correcting P values using the Benjamini-Hochberg FDR (<0.05).

Supplementary Material S8

Supplementary Figure S2 Microglia analysis at 24 h after OGD. After treatment, microglia were incubated under normoxic conditions for 24 h before cells were analyzed via western blot and supernatant was analyzed via IL-1β ELISA. **(A-B)** Protein expression of cleaved caspase-1 and cleaved IL-1β. *N = 1-4 BR/6-17 TR*. **(C)** Exemplary western blot bands of cleaved caspase-1, cleaved IL-β and β-actin comparing the treatment groups. Uncut membranes in the supplementary material, indicating representative bands. **(D)** IL-1β in microglia supernatant. *N = 5*. CTR = control, CC = control + 1 μM Crid3, OGD = oxygen-glucose-deprivation, OC = OGD + 1 μM Crid3, LPS = 6 h treatment with 100 ng/ml lipopolysaccharide. Bars display the median and the interquartile range. Statistics: Kruskal-Wallis-test (*p-values* ≤ *0.05*): (A) H_5 = 2.901; (B) H_5 = 4.690; (D) H_5 = 10.80.

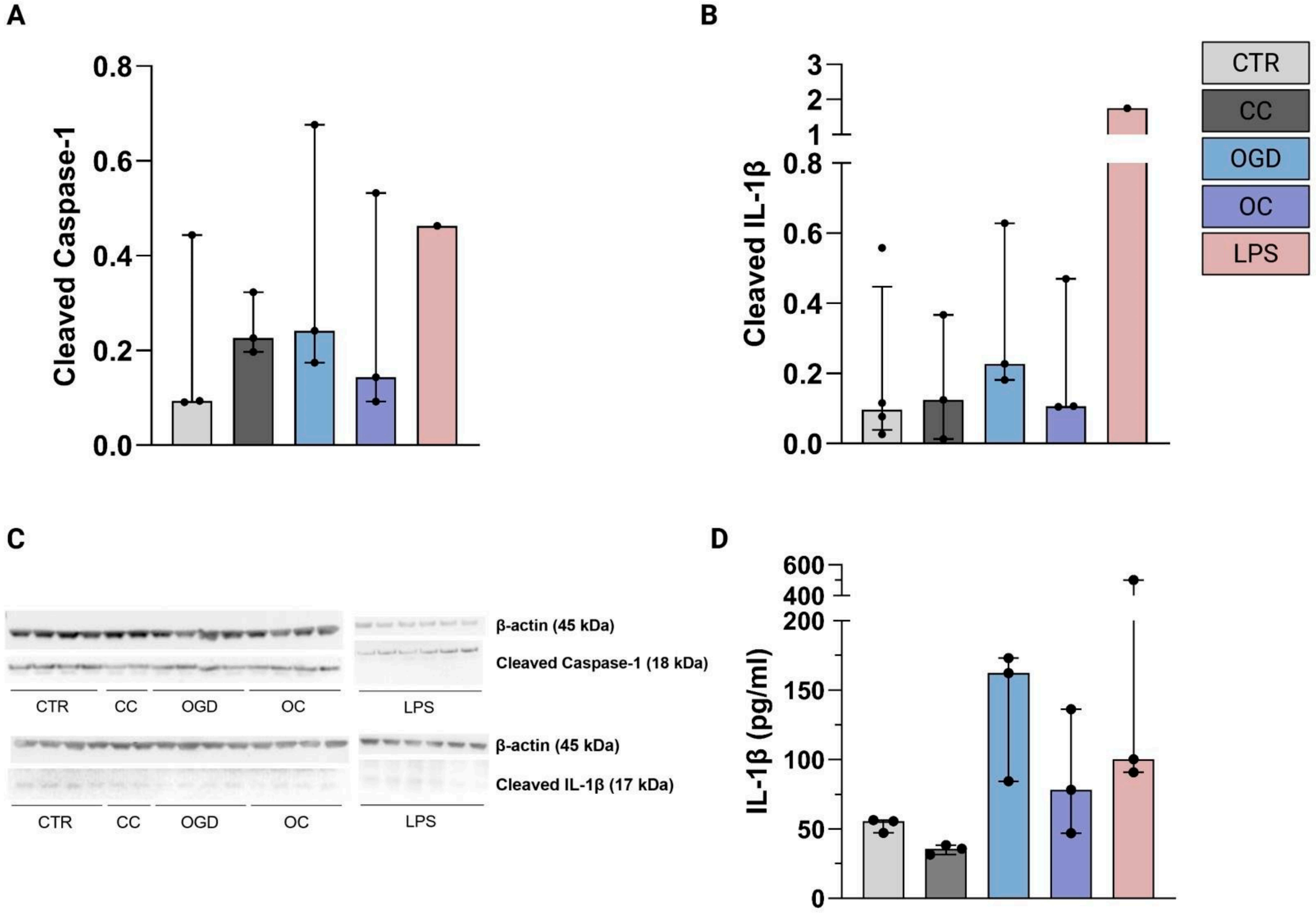

Supplementary Material S9
Supplementary Table S6 Protein genes specific to NLRP3 activation after OGD.

Gene-symbol	OGD vs. CTR		OGD vs. OC		OC vs. CTR	
	p-value	log2FC	p-value	log2FC	p-value	log2FC
Rpl24	0.018683581	5.100688711	0.085549439	3.633351547	0.476787687	1.467337164
Hnrnpab	0.081477953	4.032217221	0.258186033	2.571540821	0.517163816	1.4606764
Ccl12	0.164852362	3.448383235	0.399731456	2.065600263	0.571463975	1.382782972
Nelfe	0.053954821	3.97148724	0.197932707	2.599154569	0.491588339	1.37233267
Ufd1	0.126808671	3.614563342	0.308323582	2.380989014	0.594896838	1.233574328
Ccdc66	0.029548276	4.851344908	0.092053936	3.684695527	0.584370442	1.166649381
Lgalsl	0.086458624	4.034060905	0.19038318	3.043814246	0.66538771	0.990246659
Dtd1	0.167258042	3.574358628	0.313182021	2.587337724	0.69797689	0.987020905
Snrpd3	0.206497941	3.105520181	0.384148826	2.121757934	0.684836743	0.983762246
Ptprz1	0.217207087	2.978507735	0.40195606	2.006662872	0.683226023	0.971844863
Psme2	0.039117019	4.704545128	0.096115113	3.73841798	0.659103042	0.966127148
Rbm17	0.096630056	3.757322656	0.207870919	2.815119669	0.669076954	0.942202987
Psma8	0.179953871	3.708129279	0.313253318	2.767174063	0.729378157	0.940955216
Capzb	0.095487963	4.587357175	0.180625216	3.647912994	0.725942538	0.939444181
Smndc1	0.008637202	5.490197214	0.026289263	4.553266279	0.631780639	0.936930935
Adam10	0.058629681	4.464566928	0.129900407	3.530119127	0.682224218	0.934447801
Chmp2a	0.006078048	6.097412234	0.017469017	5.181411955	0.657232534	0.916000279
U2af2	0.095844505	2.882080362	0.206727679	2.159582515	0.668400432	0.722497847
Dstn	0.199131909	2.817162535	0.334686199	2.101586658	0.740531331	0.715575876
Psmb3	0.238506515	3.122782935	0.338530585	2.523699088	0.818763846	0.599083847
Tmod2	0.054161525	4.961942167	0.087594154	4.367187823	0.810844906	0.594754344
Arhgdia	0.128240068	3.1888548	0.202387096	2.654250595	0.794293048	0.534604205
Sh3pxd2a	0.010931615	4.980630301	0.021352721	4.450846995	0.772863233	0.529783306
Apex1	0.188267141	2.901639644	0.277959341	2.37951128	0.809785734	0.522128363
P4hb	0.255728519	2.507009164	0.347701701	2.063000196	0.838518195	0.444008968
Asah1	0.205823195	3.015360317	0.270241578	2.61773336	0.865450197	0.397626957
Uchl1	0.302223659	2.410379513	0.384548844	2.025400171	0.867787231	0.384979342
Mrpl47	0.240864522	3.3565063	0.282727122	3.067537761	0.918485999	0.288968539
Glo1	0.132194965	3.583180286	0.161518528	3.32187657	0.910612371	0.261303716
Usp39	0.016230126	5.197576545	0.019136394	5.051152873	0.942805347	0.146423672
Fkbp1a	0.174934943	2.622140698	0.195477991	2.498263324	0.947967459	0.123877374
Lfng	0.207362767	2.917673905	0.225347864	2.802293828	0.959605627	0.115380077
Ss18	0.408464124	2.222373939	0.429512304	2.122805771	0.970259066	0.099568168
Rbm28	0.080608907	4.363925638	0.084821496	4.300937476	0.979262316	0.062988162
Ppp1r11	0.341965811	2.339596365	0.343763116	2.330745707	0.997106027	0.008850659
Naga	0.36041775	2.279336468	0.352523269	2.317700307	0.987616843	-0.038363839
Gapdh	0.235846935	2.744823743	0.224766278	2.81276938	0.976263292	-0.067945637
Chd1	0.200764036	2.302711819	0.171896084	2.46448159	0.92722532	-0.161769771
H1-0	0.310244396	2.524526423	0.269480786	2.752485011	0.926258738	-0.227958588
Cd2bp2	0.007923133	5.969137258	0.005790447	6.239216803	0.897401165	-0.270079544
Rbm5	0.355611645	2.253074798	0.298685315	2.540465783	0.905426942	-0.287390985
Grb2	0.294099847	2.481999368	0.222620582	2.896232062	0.859541565	-0.414232694
Bcl11b	0.424757857	3.575429556	0.370201958	4.026527901	0.918839821	-0.451098345
Arpc3	0.044606427	5.084027261	0.027078395	5.64390548	0.818005549	-0.559878219
Sfpq	0.209671004	2.399860261	0.120152324	2.998143695	0.750998159	-0.598283433
Rpl27a	0.39799059	2.104964594	0.27300925	2.743108683	0.796501438	-0.638144088
Aoc3	0.109345413	2.807385334	0.047887031	3.51611493	0.678937846	-0.708729596
Srsf2	0.120086957	2.559818803	0.046476975	3.330167826	0.632624925	-0.770349023
Ywhah	0.298889871	2.14697487	0.151378619	2.994084272	0.679193272	-0.847109403
Srsf9	0.395789111	2.104659952	0.226717905	3.018185744	0.710856759	-0.913525791
Top2a	0.020491554	5.420826406	0.007234646	6.402790293	0.658448419	-0.981963887
Caskin2	0.180933049	3.062291647	0.0750353	4.131319582	0.635261349	-1.069027935
Crip1	0.306058496	2.099083186	0.075016115	3.729235013	0.424809546	-1.630151828
Usp5	0.410352582	2.019523335	0.094457873	4.189610617	0.376830899	-2.170087282
Krt84	0.419745024	2.306408626	0.058499059	5.568168754	0.25682009	-3.261760128
Akr1b1	0.41073413	2.293151724	0.038100629	5.994025074	0.188895191	-3.70087335
Tollip	0.156863976	3.106007839	0.002915943	7.001887168	0.078980022	-3.895879329

Supplementary Material S10

Supplementary Figure S3 Gene ontology analysis of synthesized proteins specific to NLRP3 activation after OGD. Primary microglia were exposed to 6 h of OGD (0.1% O_2). Cells were treated with 1 µM Crid3 30 min before OGD to inhibit NLRP3 (OC). During 24 h of reoxygenation, AHA labeling was performed to mark newly synthesized proteins. Supernatant was collected and prepared for MS analysis. Gene ontology analysis was performed using string-db.org.

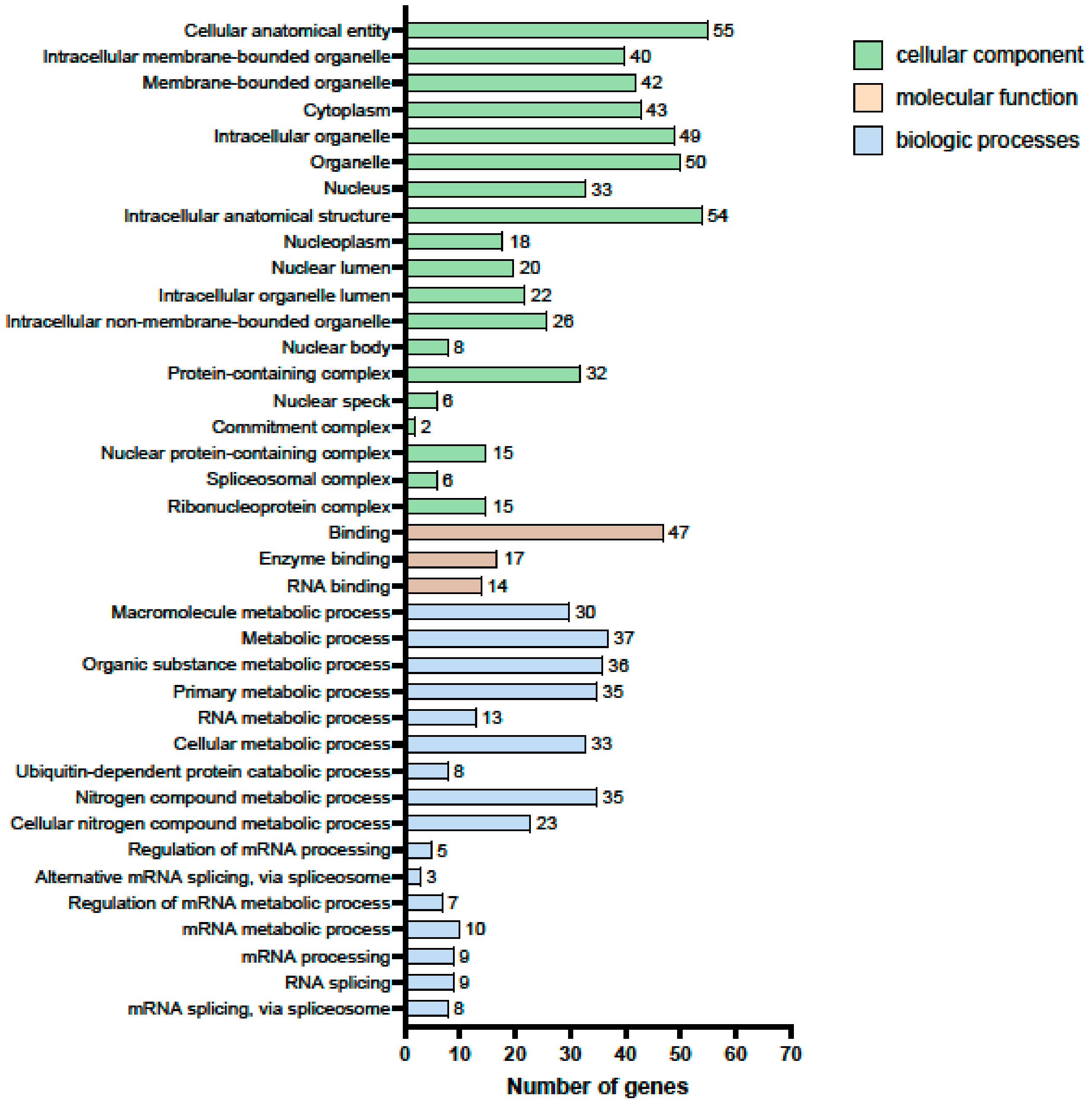

Supplementary Material S11

Supplementary Figure S4 Gender-specific effect of NLRP3 inhibition on long-term outcome in the animal. Treatment groups: Sham, HI/Vehicle and HI/Crid (5 mg/kg Crid3). (A-B) Animals were tested using Catwalk test for motor function assessment at P48 (A) and novel object recognition (NOR) for cognitive function at P76 (B). (C) Brain area loss (%) of animals at P14 and P81. Bars represent the median and the interquartile range. Males and females are separated with a line. *N (Sham) = 5, N (HI/Veh, HI/Crid) = 12*. Statistics: Kruskal-Wallis-test *(* $p \leq 0.05$)*: (A) female: H_3 = 0.6945/0.7736/0.2198/1.723/0.6593/1.459; (A) male: H_3 = 6.079/6.076/6.391/7.238/5.521/5.786; (B) female: H_3 = 3.137/4.564; (B) male: H_3 = 1.851/1.658; (C) female: H_3 = 6.780/8.357; (C) male: H_3 = 6.924/5.820.

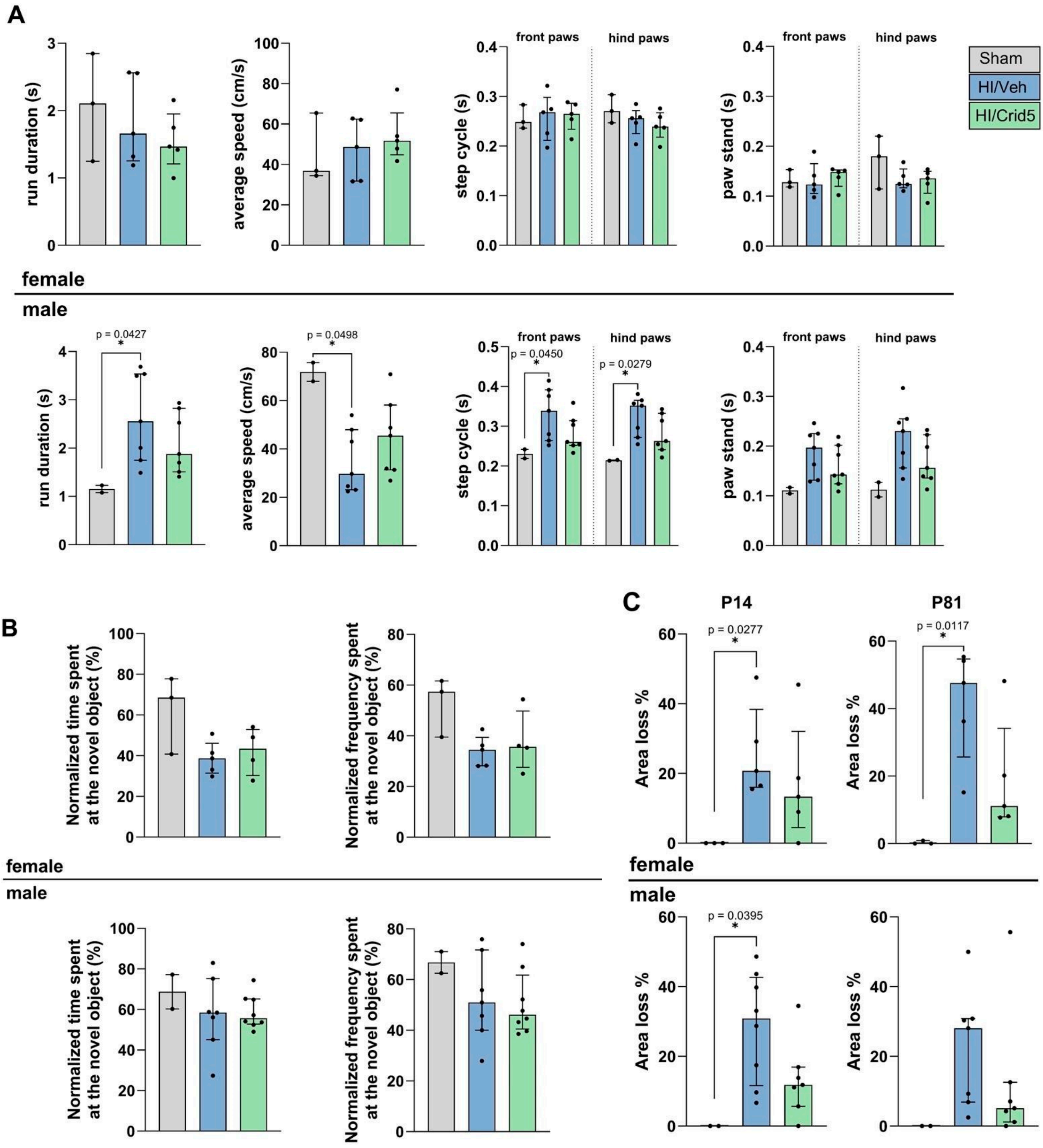

Supplementary Material S12

Western Blot Gels of Figure 3.

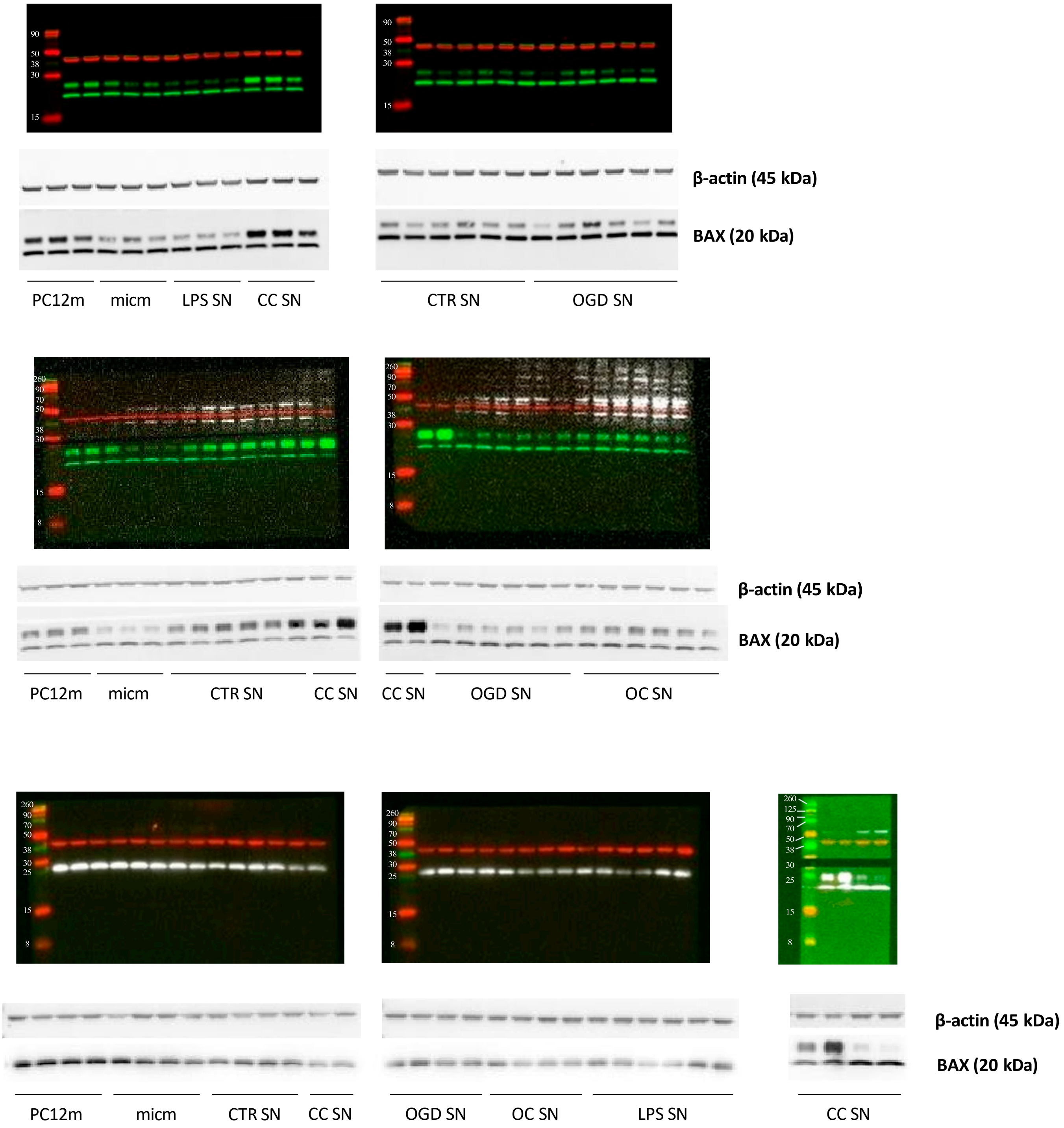

Western Blot Gels of Figure 3.

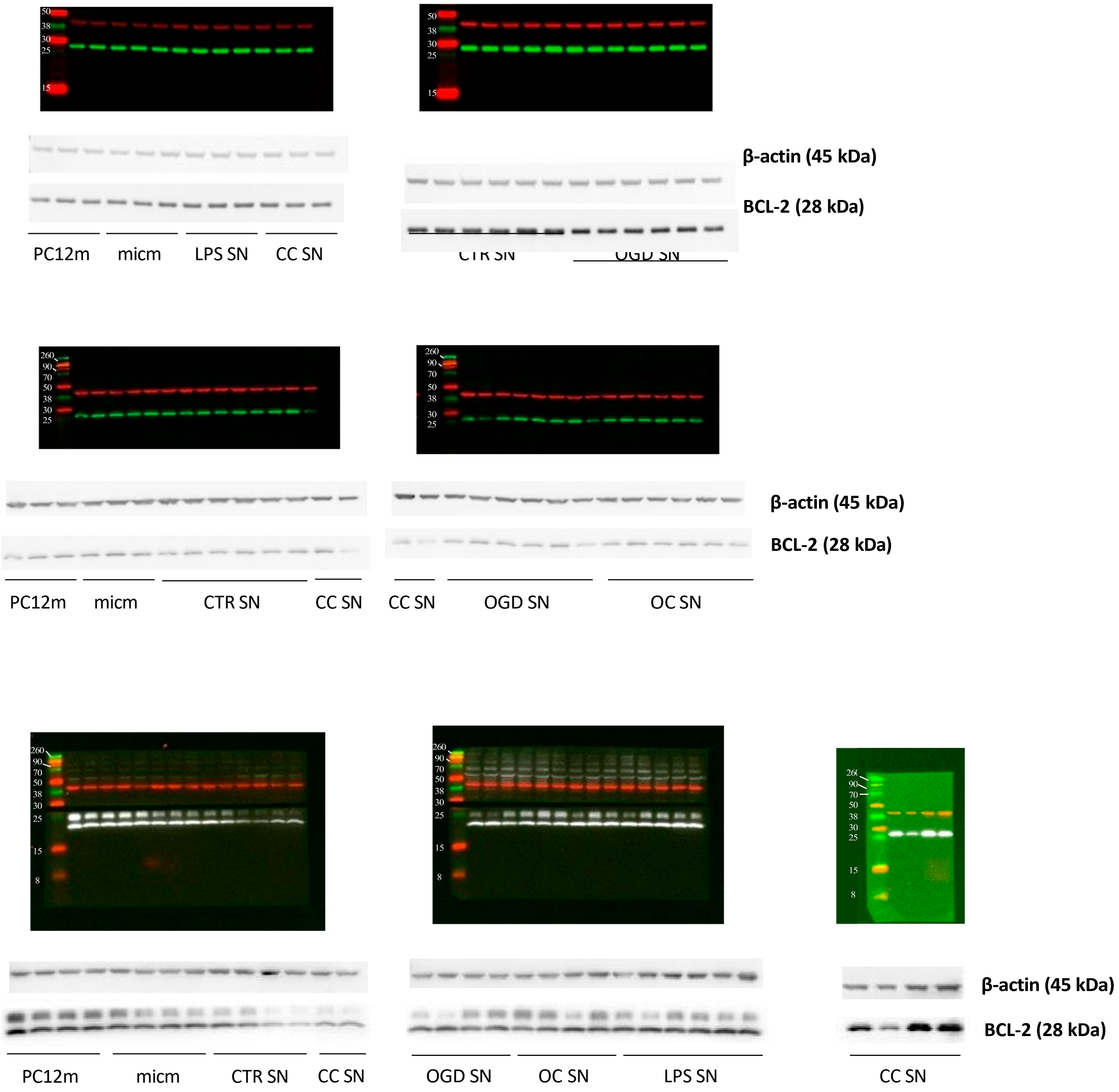

Western Blot Gels of Figure 3.

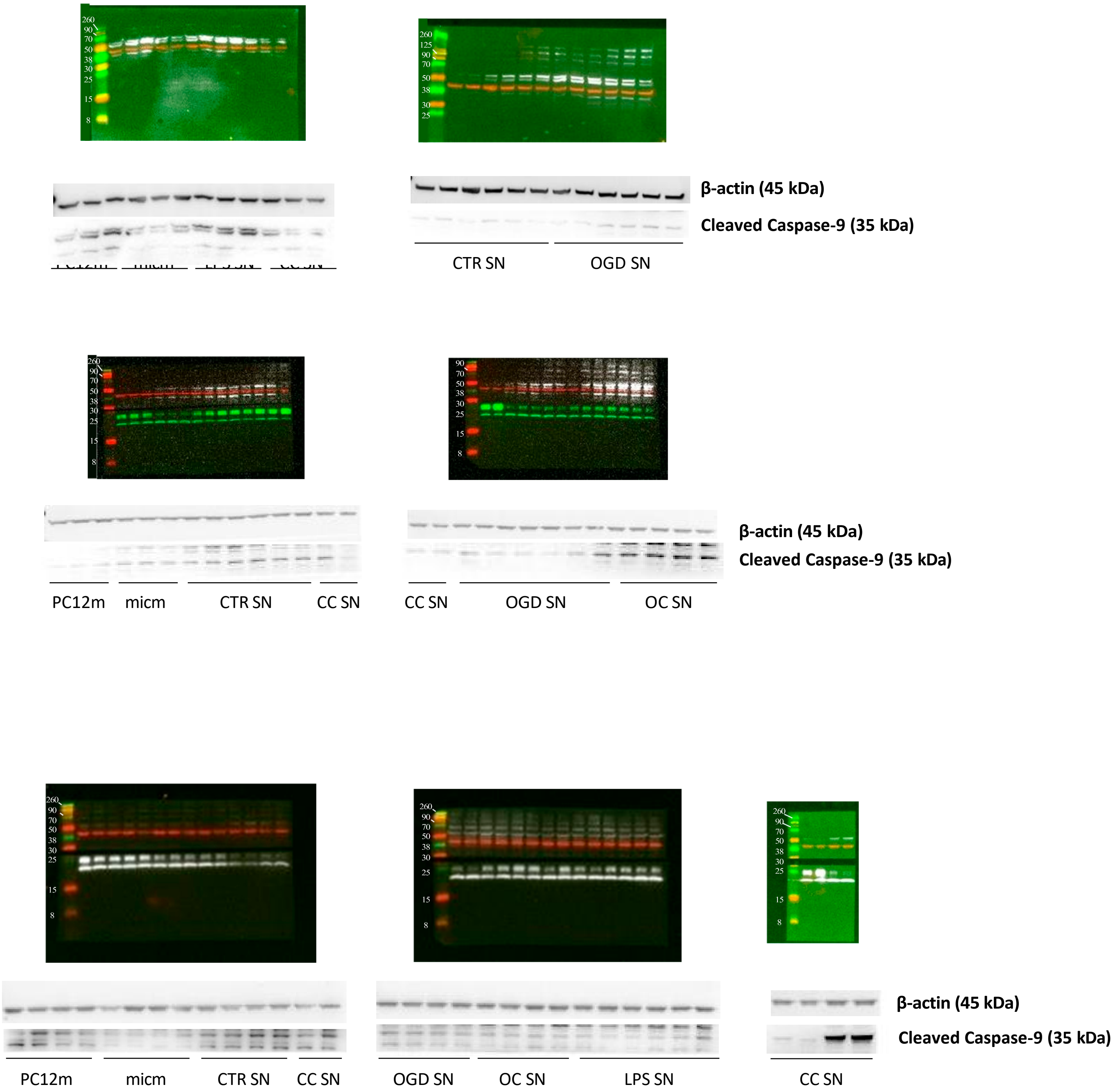

Western Blot Gels of Figure 3.

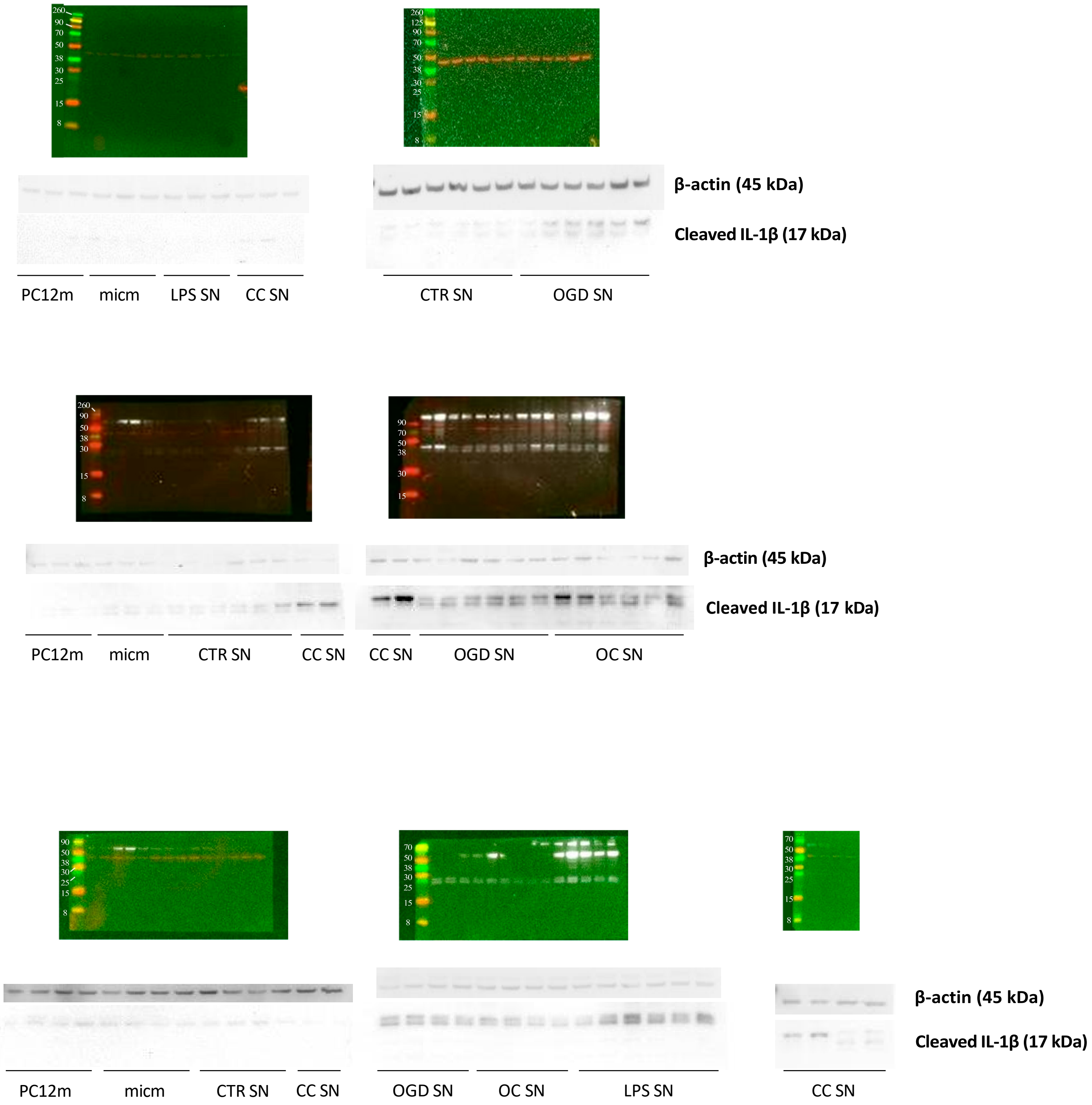

Western Blot Gels of Supplementary Figure S2.

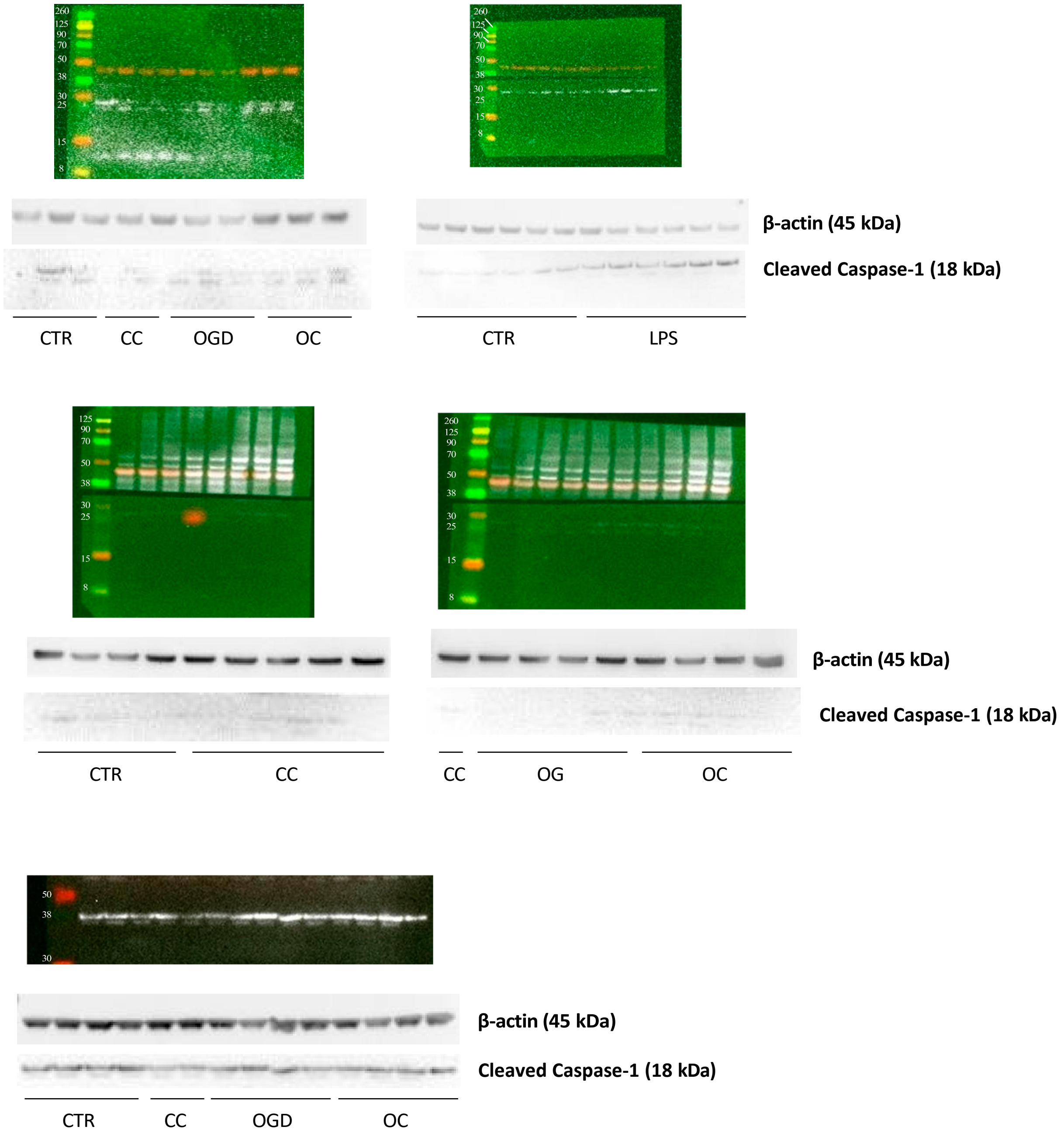

Western Blot Gels of Supplementary Figure S2.

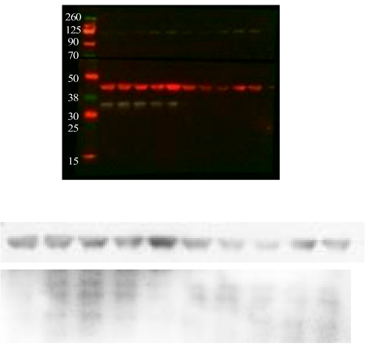

CTR CC OGD OC

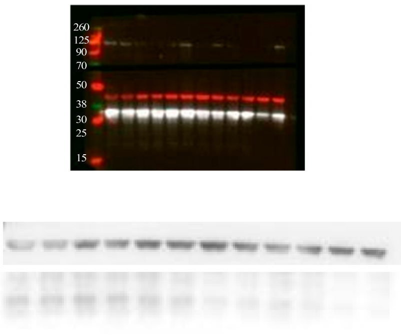

β-actin (45 kDa)

Cleaved IL-1β (17 kDa)

CTR LPS

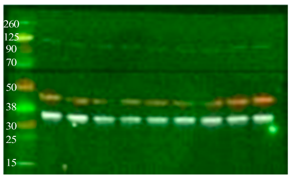

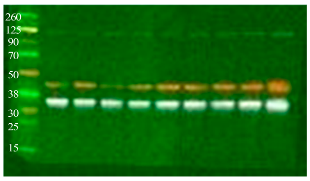

β-actin (45 kDa)

Cleaved IL-1β (17 kDa)

CTR CC CC OG OC

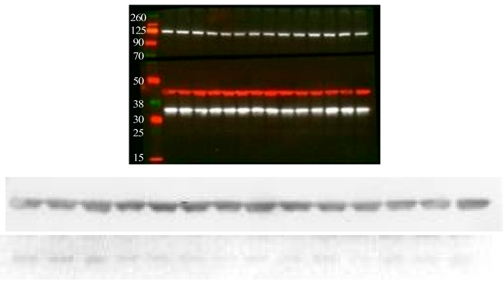

β-actin (45 kDa)

Cleaved IL-1β (17 kDa)

CTR CC OGD OC

Western Blot Gels of Figure 5 and 7 (Cortex/injection: 30 min before + 24 h after HI).

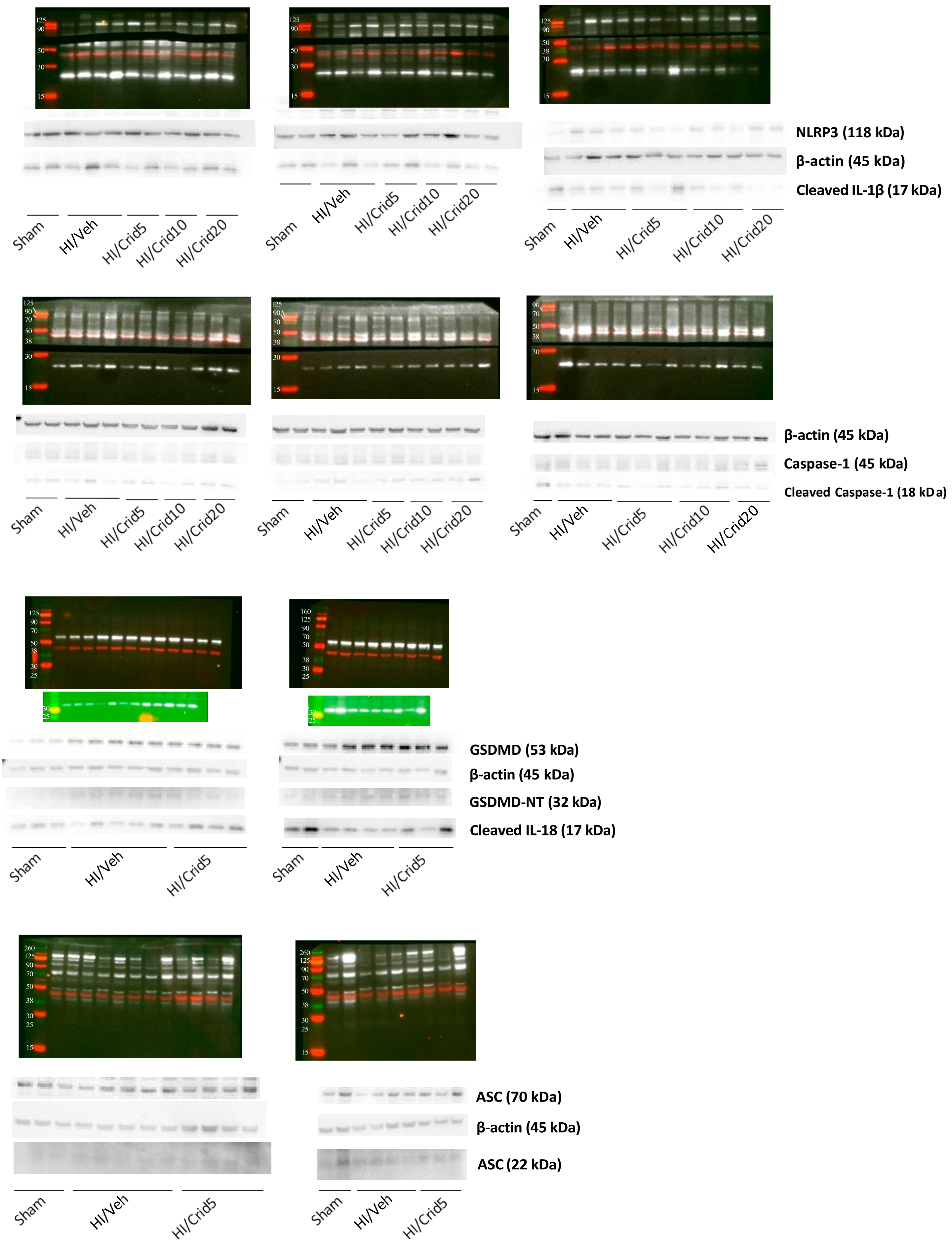

Western Blot Gels of Figure 5 (Cortex/injection: 1 h + 24 h after HI).

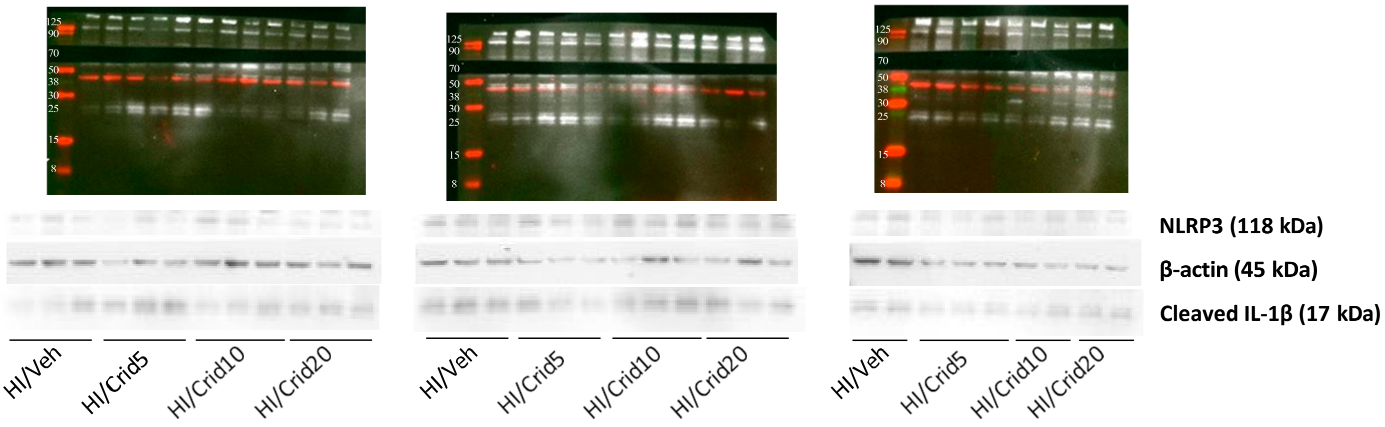

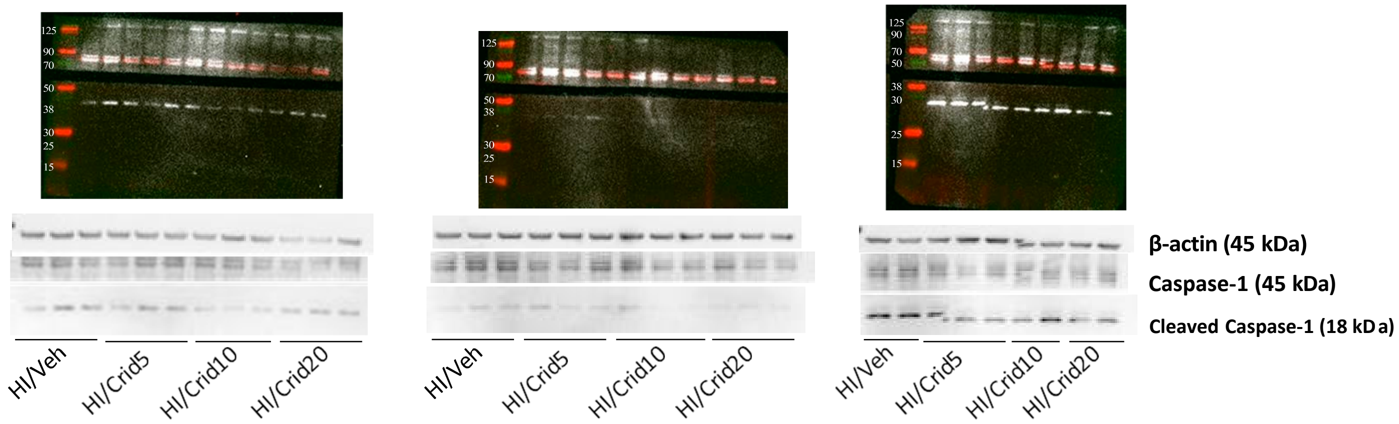

Western Blot Gels of Figure 6 (Cortex/injection: 30 min before + 24 h after HI).

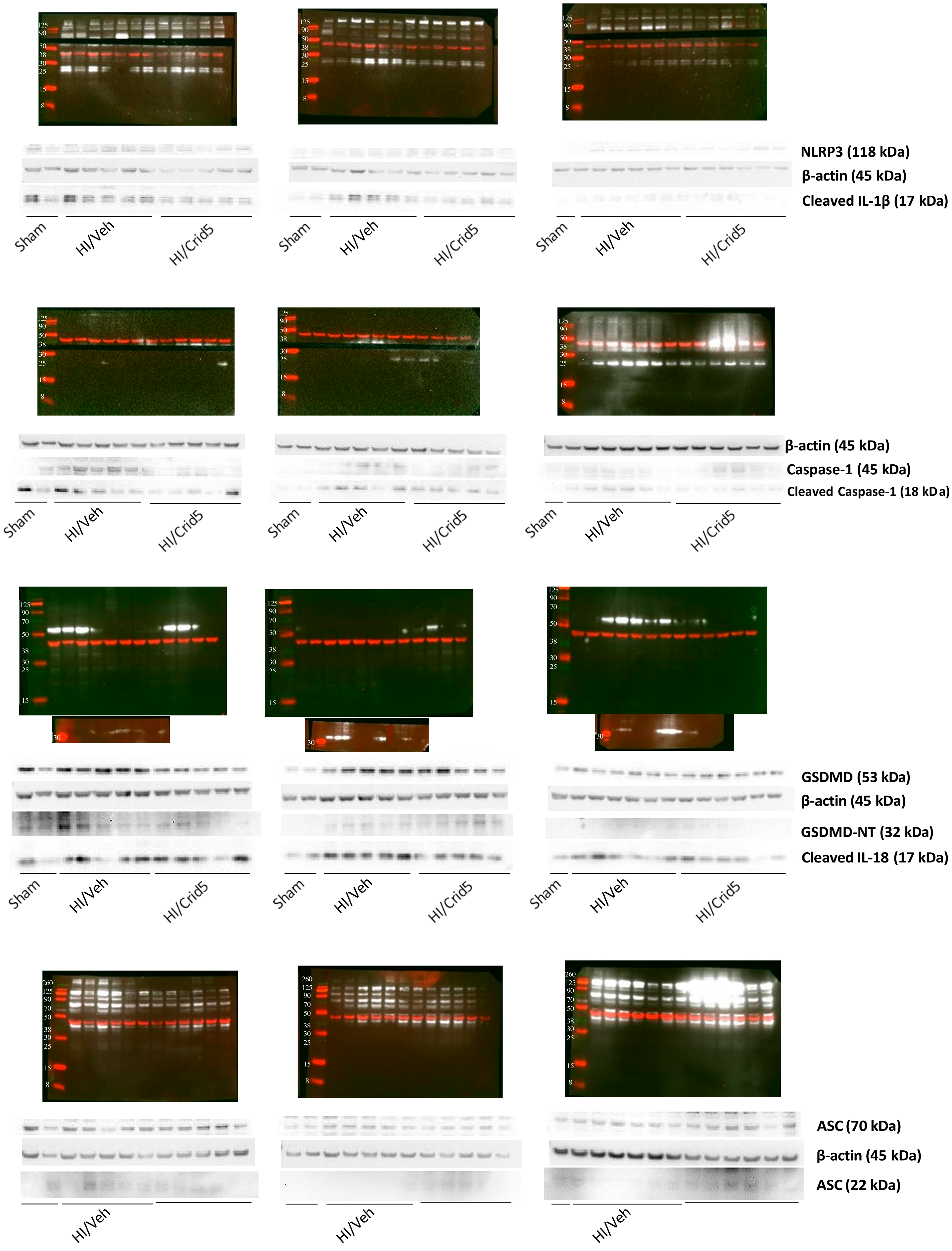

Western Blot Gels of Figure 6 (Hippocampus/injection: 30 min before + 24 h after HI).

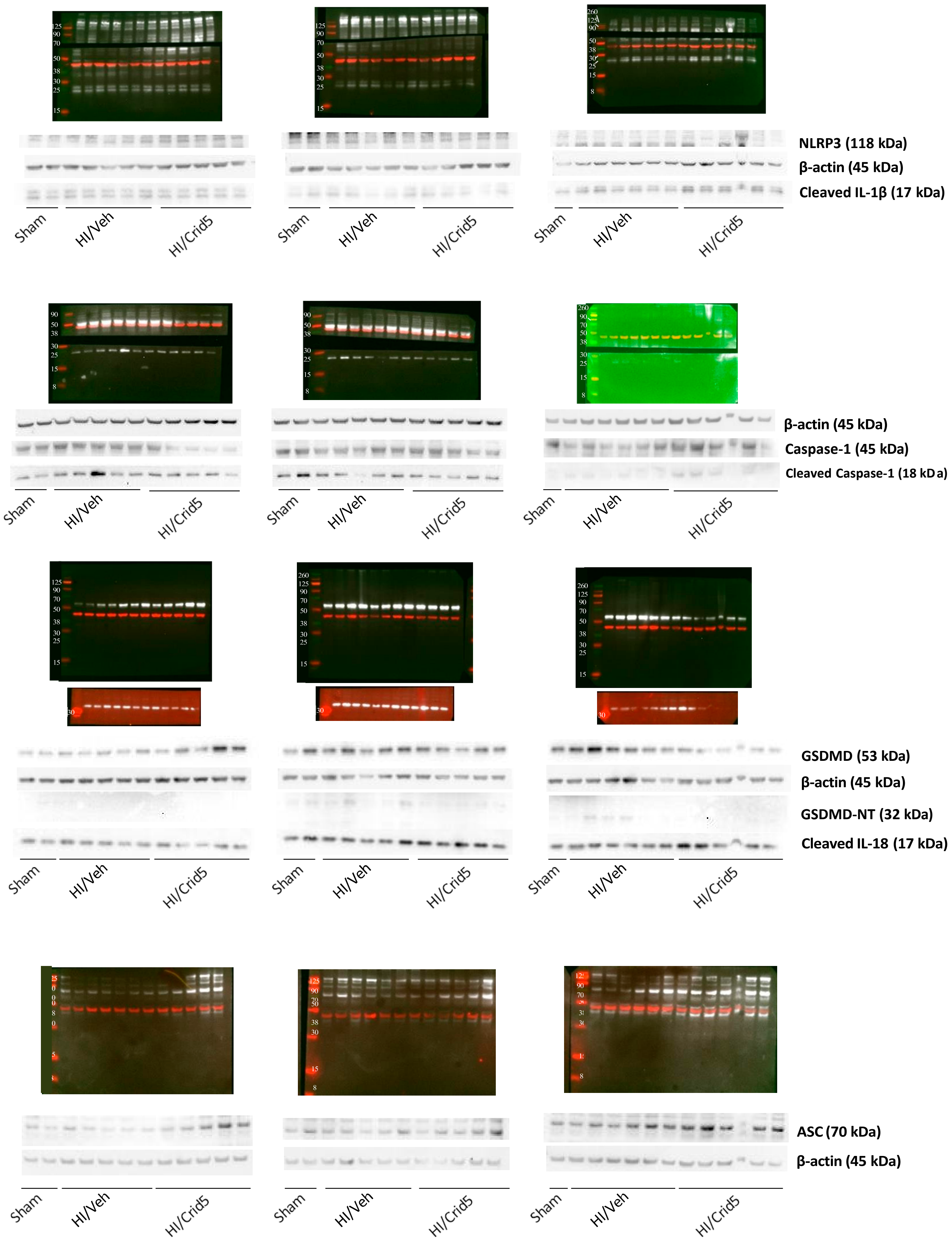

Western Blot Gels of Figure 7 (Hippocampus/injection: 30 min before + 24 h after HI).

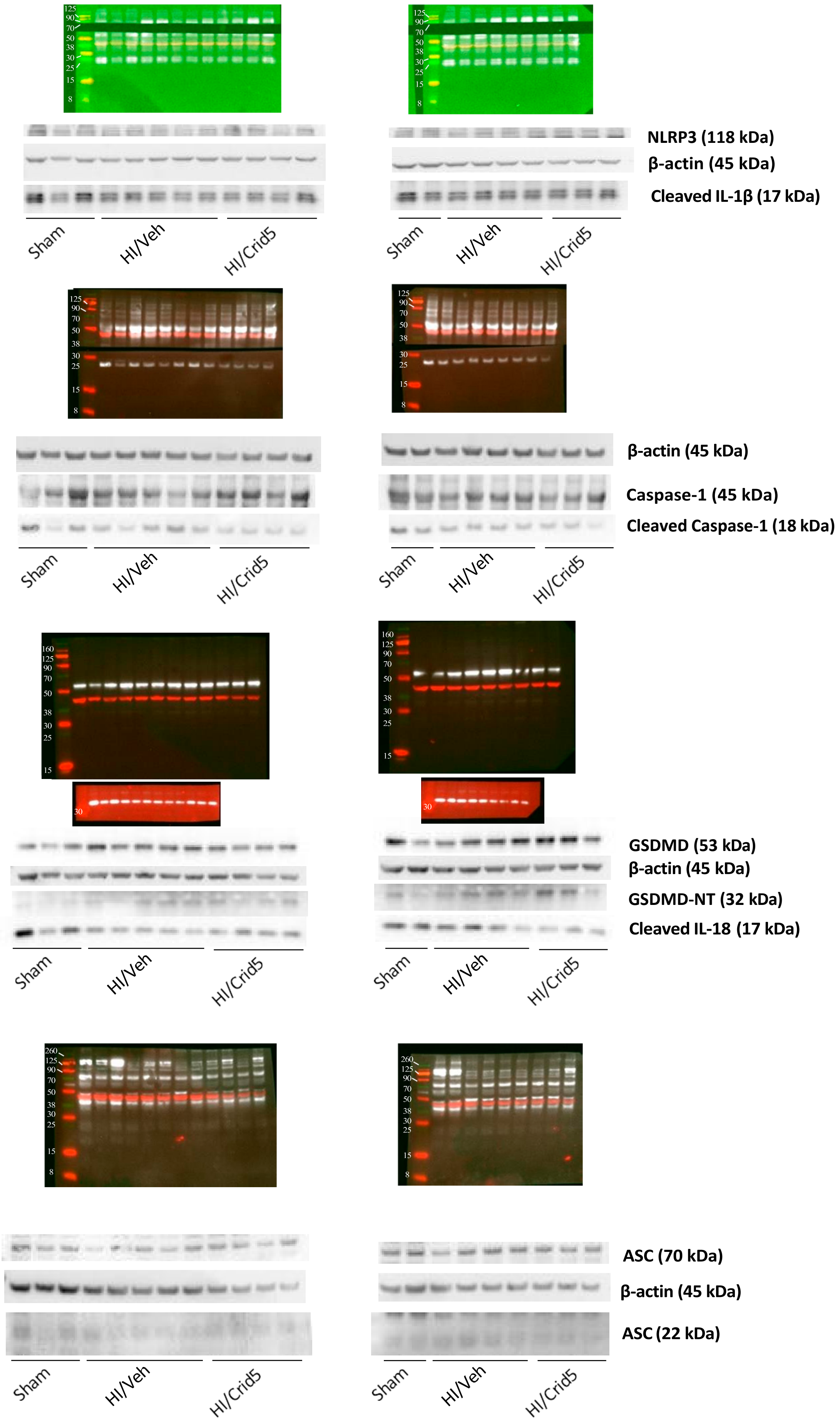

Supplement: Supplementary file 1 [file ijms-27-07853-s001.zip › ijms-4436718-supplementary.pdf]
